# CDKN2A deletion is associated with immune desertification in diffuse pleural mesothelioma

**DOI:** 10.1186/s13046-025-03522-4

**Published:** 2025-08-28

**Authors:** Federica Torricelli, Benedetta Donati, Veronica Manicardi, Mila Gugnoni, Francesca Reggiani, Gloria Manzotti, Pierluigi Di Chiaro, Cristian Ascione, Simonetta Piana, Riccardo Valli, Roberto Piro, Massimiliano Paci, Nicola Facciolongo, Filippo Lococo, Alessia Ciarrocchi

**Affiliations:** 1 Laboratory of Translational Research, Azienda Unità Sanitaria Locale-IRCCS di Reggio Emilia, Reggio Emilia, Italy; 2 Pathology Unit, Azienda Unità Sanitaria Locale-IRCCS di Reggio Emilia, Reggio Emilia, Italy; 3 Pneumology Unit, Azienda Unità Sanitaria Locale-IRCCS di Reggio Emilia, Reggio Emilia, Italy; 4 Thoracic Surgery Unit, Azienda Unità Sanitaria Locale-IRCCS di Reggio Emilia, Reggio Emilia, Italy; 5 Thoracic Surgery Unit, IRCCS-Fondazione Policlinico Gemelli, Roma, Italy; 6 Catholic University of the Sacred Heart, Roma, Italy

**Keywords:** Diffuse pleural mesothelioma, Tumor immune microenvironment, Genetic alteration, CDKN2A, Immunotherapy

## Abstract

**Introduction:**

Diffuse Pleural Mesothelioma (DPM) is a rare and incurable cancer. Immune checkpoint inhibitors (ICIs) marked some advances but only for a limited fraction of patients. Improving response prediction to ICIs is currently a clinical need in DPM. Deletion of CDKN2A gene, in chr9p21.3, is one of the most frequent alterations in DPM. As in other settings, deletion of CDKN2A locus has been associated with an immunosuppressive phenotype. Here we investigated the consequences of CDKN2A deletion (CDKN2Adel) on the tridimensional organization and function of immune infiltrate in DPM.

**Methods:**

A retrospective cohort of 89 DPMs was analyzed and assessed for CDKN2Adel through digital droplet PCR. Immune-profiling was assessed by analyzing 770 immune-related genes by digital profiling. Finally, morphologically resolved, high-dimensional transcriptomic approach was used to reconstruct the spatial architecture of immune-tumor interaction in wild-type and deleted FFPE samples.

**Results:**

CDKN2Adel was detected in 41.5% of DPMs and was associated with reduced survival (*p* = 0.04). Bulk gene expression identified 373 differentially expressed genes, of which 98.6% were downregulated in CDKN2Adel samples. These genes were enriched in several immune categories, suggesting significant immune deprivation in deleted tumors. Deconvolution analysis confirmed a major depletion of infiltrating immune cells including effector populations. Spatial transcriptomics revealed that this immunosuppressive phenotype was different according to histotype and prominent in the sarcomatoid lesions.

**Conclusion:**

These data demonstrated that CDKN2Adel deeply affects the spatial organization of immune microenvironment by depleting immune-signaling and reducing or preventing immune infiltration, supporting the potential implementation of this alteration as ICIs predictive biomarker in DPM.

**Supplementary Information:**

The online version contains supplementary material available at 10.1186/s13046-025-03522-4.

## Introduction

Diffuse Pleural Mesothelioma (DPM) is a rare and incurable cancer. Being associated with asbestos exposure, its incidence is increasing in many industrialized countries, as well as in developing areas where this silicate is still diffuse [1, 2]. DPM is a heterogeneous disease that escapes the classical genetic model of cancer evolution, and it is characterized by high molecular complexity and phenotypical plasticity [3–5]. Genomics profiling revealed a low mutational burden with a mean of < 2 somatic non-synonymous mutations per megabase and few recurrent gene mutations hitting in particular genes with oncosuppressive functions. Conversely, DPM genome displays severe chromosomal rearrangements, linked to a frequent pattern of chromothripsis or chromoplexy [6–8]. This complexity contributes to defining a high degree of heterogeneity and severely complicates the definition of DPM-oriented targeting strategies.

The introduction of immune checkpoint inhibitors (ICIs) has been regarded as a decisive turning point for this disease [1, 9]. However, results of the first clinical trials were controversial and the effects of these therapies were arguable and limited to a small fraction of patients [10–14]. Standard predictive criteria for immunotherapy eligibility in other tumors (including TMB, PD1 and PDL1 baseline expression) are not effective predictors in DPM [11, 15, 16], underlying a highly peculiar organization of the tumor immune microenvironment (TiME) in this disease. Defining strategies to boost immunotherapy currently represents one of the best options to change expectancy and quality of life in DPM patients.

DPM is an inflammatory disease since asbestos deposition causes the accumulation of reactive derivatives [17]. Inflammation deeply modifies the pleural microenvironment affecting tumor cell behavior and modulating immune signaling [18, 19]. This creates a complex and dynamic ecosystem in which DPM and immune cells affect each other in a circular and vicious crosstalk [20].

The genetic asset of cancer cells may significantly contribute to these dynamics. Chromosomal abnormalities contribute to the expression of neoantigens that stimulate immune effectors, triggering the activation of immune response [21]. Besides, many genetic alterations can influence immune regulatory signals at the transcriptional level, leading to changes in the architecture and function of the TiME [22–24].

Together with inactivating alterations of BAP1 (which are detected in up to 60% of DPMs), deletion of CDKN2A (CDKN2Adel) within the chr9p21.3 locus is one of the most frequent genetic alterations in DPM, with an overall frequency of over 40%. It has been correlated with aggressive clinical behavior and bad prognosis [5, 8, 25]. In other tumor settings, CDKN2Adel has been associated with pleiotropic phenotypical effects that go beyond the expected perturbation of cell cycle [26, 27]. In particular, in several settings [28–37], CDKN2Adel has been proposed to have immune suppressive consequences associated with reduced expression of genes related to inflammation and adaptive immune response. Also in the context of DPMs several studies indicated a potential correlation between this alteration and an immunosuppressed phenotype [4, 8, 38], even if the characterization of this phenotype and of its underlying dynamics is still partial.

While the molecular mechanisms through which CDKN2Adel contributes to multiple phenotypic effects remain to be elucidated, it is expected that the involvement of adjacent genes within the CDKN2A locus takes part to the pleiotropic effect attributed to this genetic alteration. A cluster of 16 IFN-I-related genes on chromosome 9p21 is often co-perturbed (up to 30% of cases) with CDKN2Adel potentially contributing to the immune phenotype associated with this genetic alteration. No detailed characterization of the immune-phenotype associated with CDKN2Adel and different histotypes is currently available in DPM, even if multiomics profiling suggested a potential correlation between this alteration and an immune-suppressed tumor milieu.

In this study, we aimed to explore the effect of CDKN2Adel on the spatial organization and function of the immune infiltrate within the DPM microenvironment.

## Materials and methods

### Patients cohorts and study design

A monocentric, retrospective cohort of 89 DPM patients treated in our institution from 2010 to 2021 was retrieved. Inclusion criteria were age > 18 years, availability of Formalin-Fixed Paraffin-Embedded (FFPE) tumor tissues. Histological sections from all samples were independently reviewed by two pathologists (SP and RV) for classification and material adequacy.

Clinical data, pathological characteristics, and follow-up information were thoroughly reviewed and are summarized in Table 1. The study was approved by the AVEN ethical committee (131/2023/TESS/IRCCSRE – DIMPLE).

**Table 1:** Clinical features

	nCounter	DSP
	**(N = 89)**	**(N = 9)**
**Sex**		
F	18 (79.1%)	2 (22.2%)
M	68 (20.9%)	7 (77.8%)
N-Miss	3	0
**Age**		
Mean (SD) (years)	70.7 (8.5)	74.3 (6.3)
N-Miss	2	0
**Side**		
Left	30 (35.7%)	5 (55.5%)
Right	54 (64.3%)	4 (44.5%)
N-Miss	5	
**Surgery**		
Biopsy	50 (58.1%)	6 (66.7%)
Pleurectomy	36 (41.9%)	3 (33.3%)
N-Miss	3	0
**Histology**		
Epithelioid	34 (39.1%)	0
Biphasic	36 (41.4%)	6 (66.7%)
Sarcomatoid	17 (19.5%)	3 (33.3%)
N-Miss	2	0
**Stage**		
I	39 (47.6%)	5 (62.5%)
II	4 (4.9%)	0
III	7 (8.5%)	0
IV	32 (39.0%)	3 (37.5%)
N-Miss	7	1
**Asbestos exposure**		
Yes	77 (94.4%)	4 (100%)
No/uncertain	4 (5.6%)	0
N-miss	18	5
**Survival status (2 years FU)**		
Alive	22 (26.9%)	1 (14.3%)
Dead	60 (73.1%)	6 (85.7%)
N-Miss	7	2
**Overall Survival**		
Median [range](months)	15.0 [2-117]	9 [2–20]
N-Miss	7	2

### Digital droplet PCR

For each DPM sample (*N* = 89), DNA was extracted by Maxwell^®^ RSC DNA FFPE Kit (Promega) starting from 2 mm³ FFPE specimens. DNA quality and quantity were evaluated by NanoDrop 2000 (Thermo Fisher Scientific). CDKN2A deletion status was assessed by digital droplet PCR (Bio-Rad Laboratories) using the CNV FAM Assay Human CDKN2A dHsaCP1000581. For IFN I locus analysis we used a custom-designed assay targeting a region localized in the middle of the IFN I locus (FAM Assay dHsaCNS635451600). EIF2C1 (CNV HEX Assay Human AGO1, UniqueAssayID : dHsaCP2500349) was used as reference control region in both analyses. Each reaction was set up starting from 10-20ng of DNA and following manufacturer’s protocol. Results were collected by QX Manager Software v2.2 (Bio-Rad Laboratories) and analyzed by QX Manager Software v2.2 (Bio-Rad Laboratories). Patients were considered deleted when presenting copies of the target genes < 1.55.

### Digital bar-coding bulk gene-expression profiling

**Fig. 1:**
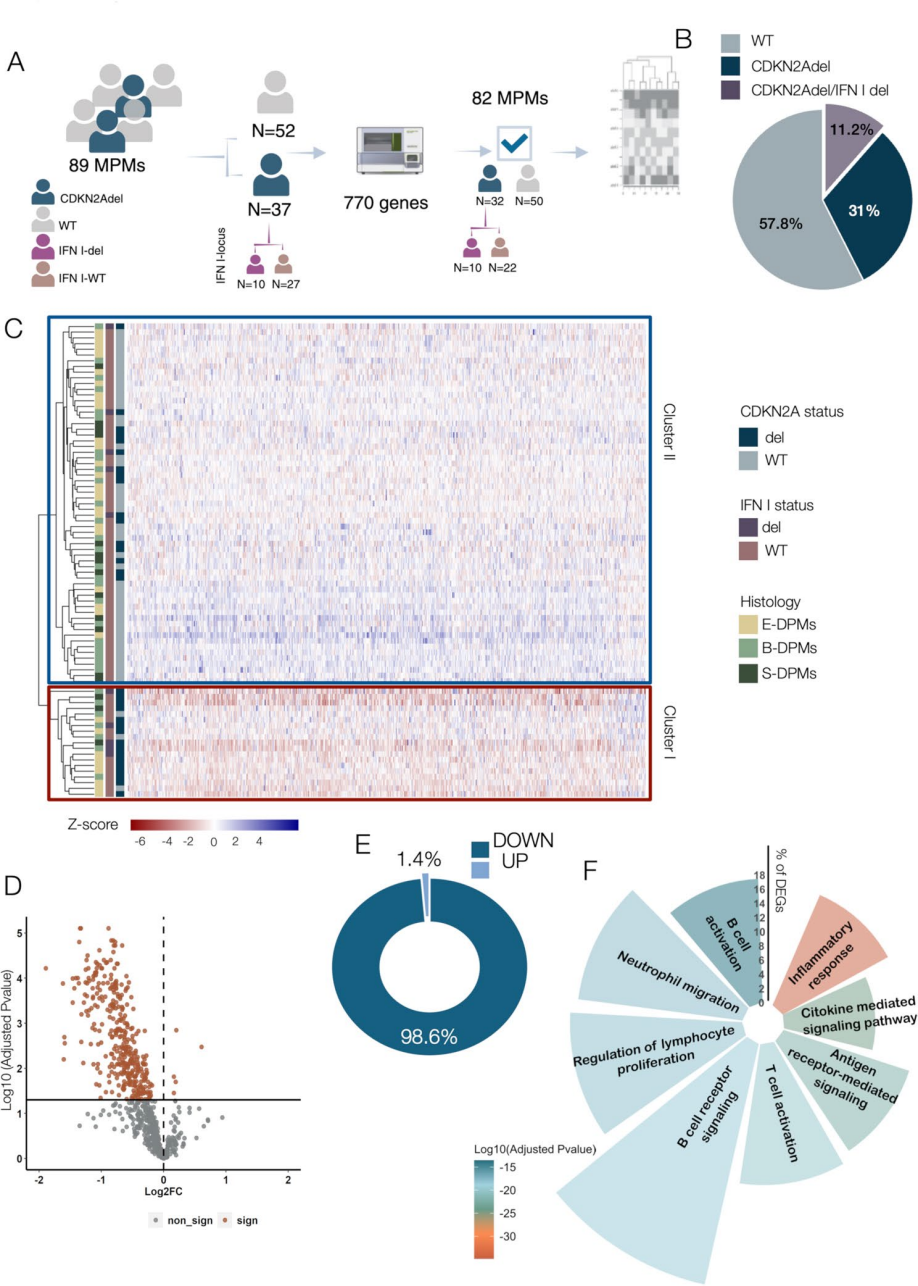
CDKN2adel associates with reduced survival probability **A**. Experimental study design. The status of CDKN2A deletion was investigated in 89 retrospective DPMs by ddPCR. 82 patients were successfully profiled. A panel of 770 immune related genes was analyzed by digital counts (nCcounter NanoString Technologies). **B**. Pie chart representing the percentage of WT, CDKN2A deleted and CDKN2Adel/IFN Idel patients in our cohort of 89 DPMs. **C**. Unsupervised clustering analysis based on gene expression on the entire cohort. Two clusters were identified. Color gradient expresses the z-score of each gene in each sample. Color tags indicate the genetic status (CDKN2A and IFN I) and histology. **D**. Volcano plot showing the expression and the Adjusted-Pvalue of DEGs in CDKN2Adel and WT DPMs. Orange dots represent significantly deregulated genes (Adjusted PValue < 0.05). **E**. Donut chart showing the percentage of up- regulated and downregulated DEGs obtained from CDKN2Adel and WT DPMs comparison. **F**. GO analysis of DEGs. Circular histograms illustrating the top scoring biological categories. For each biological category, colors gradient represents significance (Log10 adjusted PValue) while bars height represents the percentage of involved DEGs.

Bulk gene expression was conducted by nCounter ( Nanostring Technologies*)*, as previously described [39, 40]. Total RNA was extracted from five 5 μm FFPE slides from surgical specimens, using the Maxwell^®^ RSC RNA FFPE Kit (Promega) and assessed and quantified by NanoDrop 2000 (Thermo Fisher Scientific)(Fig. 1A). Gene expression was conducted using the NanoString PanCancer Immune Profiling Panel, which includes 770 immune-related genes. Data were processed with nSolver Analysis Software 4.0. After imaging quality control, raw gene counts were normalized on technical controls and housekeeping genes [40]. For each gene, fold change (FC) was calculated as the expression ratio between CDKN2Adel vs. WT samples. PValue was calculated by two-tailed Student’s t-test and adjusted by Benjamini–Hochberg method. Gene expression data are available at GEO accession number: GSE302048.

### Spatial transcriptomics

Spatial Transcriptomics was conducted using the GeoMx DSP^®^ platform (Nanostring Technologies, USA), starting from one 5 μm-thick FFPE tumor tissue section, as previously described [20, 41]. Briefly, the GeoMx^®^ Cancer Transcriptome Atlas panel was hybridized to the sections following the manufacturer’s protocol. This panel contains RNA probes targeting 1834 genes, enabling comprehensive profiling of tumor biology and the tumor microenvironment, including canonical cancer pathways, immune cell types, and checkpoint molecules. Sections were stained with the GeoMx Solid Tumor TME Morphology Kit (GMX-RNA-MORPH-HST-12; Nanostring Technologies) according to manufacturer’s instruction. Morphology and Pan-CK staining were used to distinguish the histological components and to segment Representative Regions of interest (ROIs) in specific Epithelioid (E-) and Sarcomatoid (S-)areas of illumination (AOIs). ROI were designated by two pathologists (SP, RV). An average of 22 circular AOIs per sample was selected (range: 16–30). RNA probes from selected AOIs were collected and NGS libraries were constructed by using the GeoMx^®^ Seq Code Pack (Nanostring Technologies) according to protocol. Libraries were pooled based on the AOI size, purified using AMPure XP beads (Beckman Coulter, Brea, California, USA), and resuspended in a volume of elution buffer proportional to the number of pooled AOIs. The quality and quantity of the library pools were assessed with the Agilent Bioanalyzer. Libraries were diluted to 1.6 pM and sequenced using Illumina NextSeq500 (paired-end 2 × 27), targeting a minimum coverage of 30 reads per µm² of collected area. After sequencing, FastQ files were demultiplexed and converted into DCC files via the GeoMx^®^ Next-generation Sequencing (NGS) Pipeline App in the BaseSpace Illumina Sequence Hub. These files were subsequently uploaded to the GeoMx DSP platform and mapped to their respective AOIs.

### Spatial transcriptomics data analysis

Data analysis was conducted using GeoMx DSP analysis suite. First quality check (QC) step was conducted on AOIs to verify: quality of sequencing (raw reads ≥ 1000, aligned reads ≥ 80, sequencing saturation ≥ 40%,) number of collected nuclei (> 100), background effect (negative probe count mean ≤ 4.5, no template control counts ≤ 100). AOIs that did not reach those thresholds were excluded from further analysis. The selected AOIs had a median surface area of 73,777 μm² (range: 19603–253227 μm²) and contained a median of 399 nuclei (range: 109–1602). The second QC step was the exclusion of low-performing probes from target counts calculation (geometric mean of probe in all segments/ geometric mean of probe within each target ≤ 1; failed Grubbs outlier test in ≤ 20% of AOIs). Then we applied a filter on AOIs, excluding the ones in which less than 20% of the genes were expressed over the LOQ ( 2 SDs above the geometric mean of negative probes). For samples that passed filters, raw gene counts were normalized on the third quartile (Q3) of all target genes according to the manufacturer's indications. Differential analysis was conducted by comparing CDKN2Adel vs. WT AOIs for each histological component. Differentially expressed genes (DEGs) were defined by linear mixed model adjusted for patients' ID. Gene Ontology (GO) enrichment analysis was performed on Reactome pathways by EnrichR online software. Significance threshold was set at adjusted PValue < 0.05 (Benjamini-Hochberg correction). Data are available at ArrayExpress accession number: E-MTAB-15,378.

### Deconvolution analysis

For the bulk nCounter dataset, cell deconvolution was performed through SpatialDecon R package (v1.12.3). The geometric mean of negative probes was calculated for each sample and used to build the background matrix required by spatialdecon function. For the spatial GeoMX-DSP dataset, cell deconvolution was performed using the “SpatialDecon” pipeline, available on GeoScript Hub (https://nanostring.com/products/geomx-digital-spatial-profiler/geoscript-hub/), and applied through the GeoMx DSP platform. For both datasets cell deconvolution was performed considering SafeTME [42] as reference matrix and results were exported as scaled abundance scores.

To validate deconvolution results on nCounter data, we repeated deconvolution analysis by CIBERSORTx (https://cibersortx.stanford.edu/) using as input the signature matrix LM22, that includes 22 immune cell types, and setting 100 permutations. Data were exported in absolute mode. Percentages for each cell type were compared between CDKN2Adel and WT AOIs. PValues were calculated applying Wilkoxon test.

### Statistical analysis

Bioinformatic and statistical analyses of gene expression profiles were conducted using R Software v4.3.1. Spearman’s test was applied for correlation analysis and two-tailed Student’s t test was used to compare single gene expression between two groups.

All correlation plots, boxplots and histograms were performed using “ggplot2” and “ggpubr” R packages. Alluvial plot, forest plot and chord plot were produced using “alluvial”,”forestplot” and “circlize” R packages, respectively. Heatmaps were generated using “Pheatmap” R package (v1.0.12).

For survival analyses, patients were dichotomized either based on genetic status (CDKN2Adel or CDKN2Adel/IFN I-del) or median gene expression and PValues were calculated using the Log-rank test. Survival plots were prepared using the “Survival” and “Survminer” R packages. Tests were considered statistically significant with a PValue < 0.05 and adjusted PValues were calculated using the “Benjamini-Hochberg” method.

## Results

### Analysis of CDKN2Adel in the study cohort

A retrospective cohort of 89 DPMs was selected (Fig. 1A). Clinical data are reported in Table 1.

41.6% (*N* = 37) of DPMs were deleted for CDKN2A, in line with previously reported frequencies [8]. Correlation of CDKN2Adel with main clinical data is reported in Supplementary Table 1 and did not show significant associations. A correlation trend, even if not non-significant, was observed between CDKN2Adel and the non-epithelioid phenotype, both in our internal cohort (Supplementary Fig. 1A) and in the TCGA-MESO dataset (Supplementary Fig. 1B). Survival analysis represented by Kaplan-Meier curves showed that CDKN2Adel was significantly associated with reduced survival probability in our cohort (Supplementary Fig. 1C) and in the TCGA dataset (Supplementary Fig. 1D). Co-deletion of a cluster of 16 IFN I-related genes on Chr 9p21 occurs in a subset of DPMs showing CDKN2Adel. Perturbation of this cluster may be relevant for the definition of the immune phenotype associated with this alteration. Deletion of IFN I locus was observed in 11.2% of the entire cohort and in 27% of CDKN2Adel samples (Fig. 1B). These frequencies are in line with those previously reported. As for CDKN2Adel alone, a correlation trend, even if not non-significant, was observed between the presence of double deletions and the non-epithelioid phenotype in our internal cohort (Supplementary Fig. 1E). However, Survival analysis showed that CDKN2Adel/IFN Idel double mutants showed a significantly reduced survival as compared with WT DPMs but no difference was observed with the curve of CDKN2Adel DPMs (Supplementary Fig. 1F) indicating that the co-deletion of IFN I cluster does not further increase the prognostic impact of CDKN2Adel alteration.

### CDKN2Adel is associated with an immunosuppressed phenotype in DPM

To analyze the effect of CDKN2Adel on TiME, we performed an immune-related gene expression profiling on FFPE samples in an internal retrospective cohort of 89 DPM samples (Table 1). 82 samples were successfully profiled. Of these, 39% (*N* = 32) were CDKN2Adel and 61% WT (*N* = 50) (Fig. 1A).

Unsupervised clustering analysis based on gene expression subdivided the cohort into two clusters. 84% of Cluster I was composed of patients deleted in CDKN2A, while Cluster II included over 75% of WT samples (Fig. 1C). A similar trend was observed in the TCGA dataset, where Cluster I was composed of up to 73% of CDKN2Adel samples (Supplementary Fig. 2A). These data indicated that immune expression profiling largely overlays the genetic subgroups. Noticeably, we did not observe a specific association of IFN I-cluster co-deletion with the identified clusters, suggesting that the most relevant effect on this clusterization was determined by the deletion of CDKN2A.

Differential analysis between CDKN2Adel and WT DPMs in our cohort identified 373 differentially expressed genes (DEGs) (Adjusted PValue < 0.05) (Fig. 1D).

Up to 98.6% of DEGs (*N* = 368) were downregulated in CDKN2Adel as compared to WT samples while only 1.4% of DEGs (*N* = 5) showed the opposite trend, indicating a deep inhibition of the immune-related signals following CDKN2Adel in DPM (Fig. 1E).

**Fig. 2:**
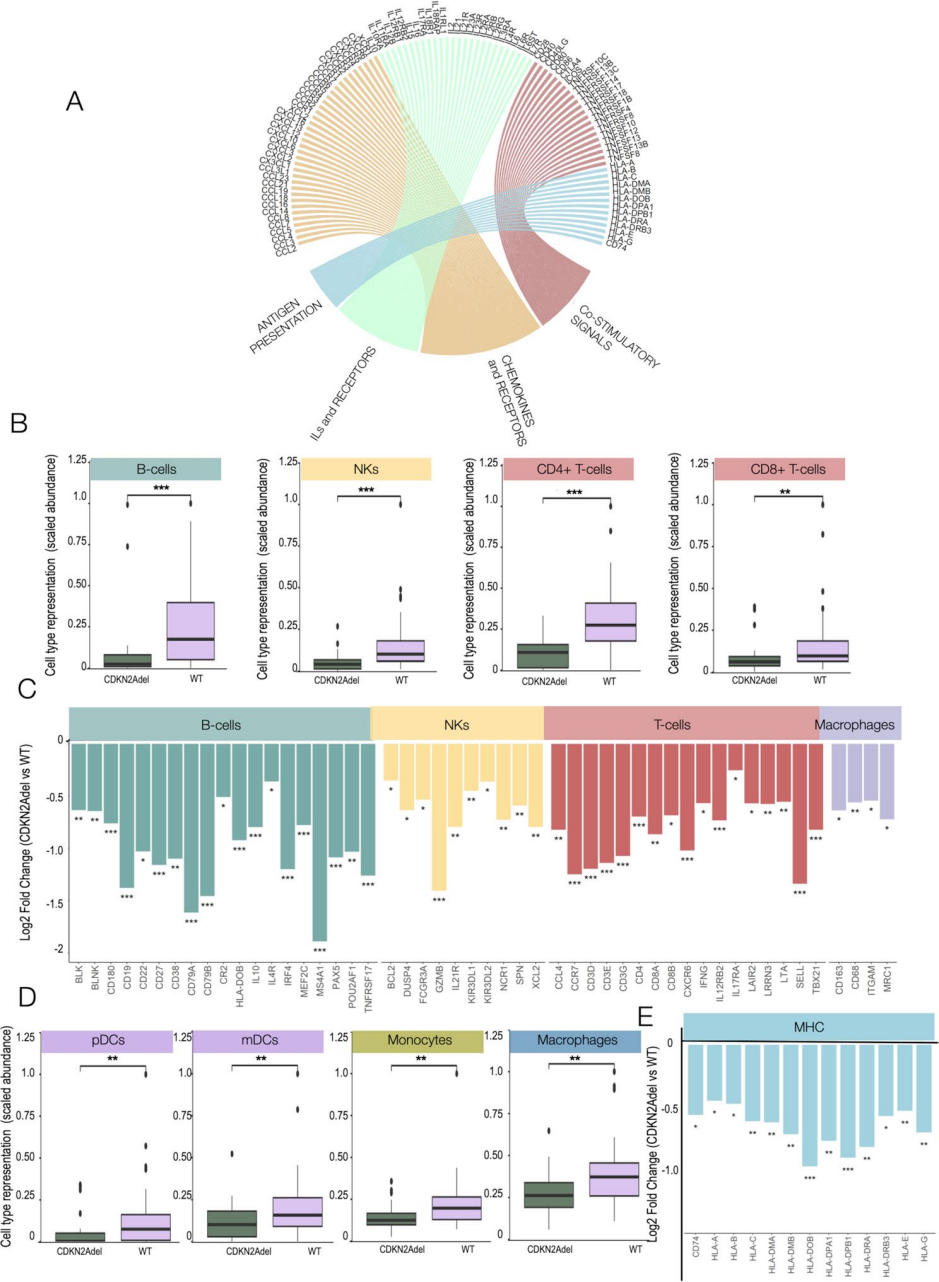
CDKN2Adel affects the immune transcriptional profile of DPM **A**. Chord plot representation of the convergence of DEGs on the indicated immune categories perturbed by CDKN2Adel. Arches are colored according to functional categories. **B**. Deconvolution analysis in CDKN2Adel versus WT DPMs. Boxplots represent the predicted scaled abundance of the indicated immune populations estimated on gene expression profiles. **C**. Bar plots report the differential expression of lineage specific markers for the indicated immune cell populations. For each gene the Y-axis represents the Log2Fold Change, in CDKN2Adel vs. WT DPMs. **D**. Deconvolution analysis in CDKN2Adel and WT DPMs. Boxplots represent the predicted scaled abundance of the indicated immune populations estimated based on gene expression profiles. **E**. Bar plots report the differential expression of MHC genes. For each gene the Y-axis represents the Log2Fold Change in CDKN2Adel vs. WT DPMs. PValue: *0.01–0.05, ** 0.001–0.01 *** <0.001

GO analysis showed that major enriched categories among CDKN2Adel downregulated DEGs were Inflammation, Cytokines mediated signaling and biological functions involved in adaptive immunity activation (Fig. 1F). Two large genes blocks emerged: (i) signaling molecules including chemokines, interleukins and their related receptors, (ii) components of the antigen presentation machinery (both MHC I-II) and accessory co-regulatory molecules (Fig. 2A), indicating a significant impairment of immune-stimulatory signals. We also performed a gene expression comparison between CDKN2Adel and CDKN2Adel/IFN Idel DPMs to explore genes specifically affected by the deletion of IFN I cluster. Only 47 genes resulted significantly deregulated in double deleted samples (Supplementary Fig. 3A), 43 of which were downregulated (91.5%) in CDKN2Adel/IFN Idel subgroup indicating a loss of function. As expected, these genes were enriched in IFN-related pathways, confirming the validity of our analysis (Supplementary Fig. 3B).

To explore the functional consequences of these transcriptional changes, we performed a deconvolution analysis based on gene expression in CDKN2Adel and WT DPM, that showed a severe depletion of the estimated relative abundance of immune effector cells (including CD4 + and CD8 + T-cells, B-cells and NKs) in deleted samples as compared to WT (Fig. 2B). Coherently, the expression of lymphoid and myeloid lineage-specific markers resulted significantly downregulated in CDKN2Adel DPMs (Fig. 2C). Consistently, in mutated samples transcriptomic profiles predicted a significant depletion of antigen-presenting cells (Fig. 2D) supported by a severe downregulation of MHC class II genes (Fig. 2E). Class I MHC molecules were also impaired further limiting adaptive immune response stimulation (Fig. 2E). These data were validated using a second deconvolution algorithm (Supplementary Fig. 4) and extended to evaluate the potential impact of IFN I cluster co deletion on this phenotype (Supplementary Fig. 5). No significant difference was observed between CDKN2Adel and CDKN2Adel/IFN Idel subgroup of samples.

Taken together, these results indicated that CDKN2Adel associates with an impaired TiME in which immune cells recruitment is inhibited by a decrease of both secreted chemotactic and cell-mediated stimulatory signaling. To complete this set of information we also checked the expression of immune checkpoint genes observing a significant downregulation of these inhibitory molecules in CDKN2Adel DPMs (Supplementary Fig. 6). This observation is coherent with the hypothesis that CDKN2Adel is associated with an immune-desert phenotype rather than with the establishment of immune exhaustion mechanisms. Also these data seem to indicate that this effect is in large part independent of the perturbation of the neighboring IFN I cluster.

#### CDKN2Adel affect macrophages chemotaxis and their relationship with immune effector cells

**Fig. 3:**
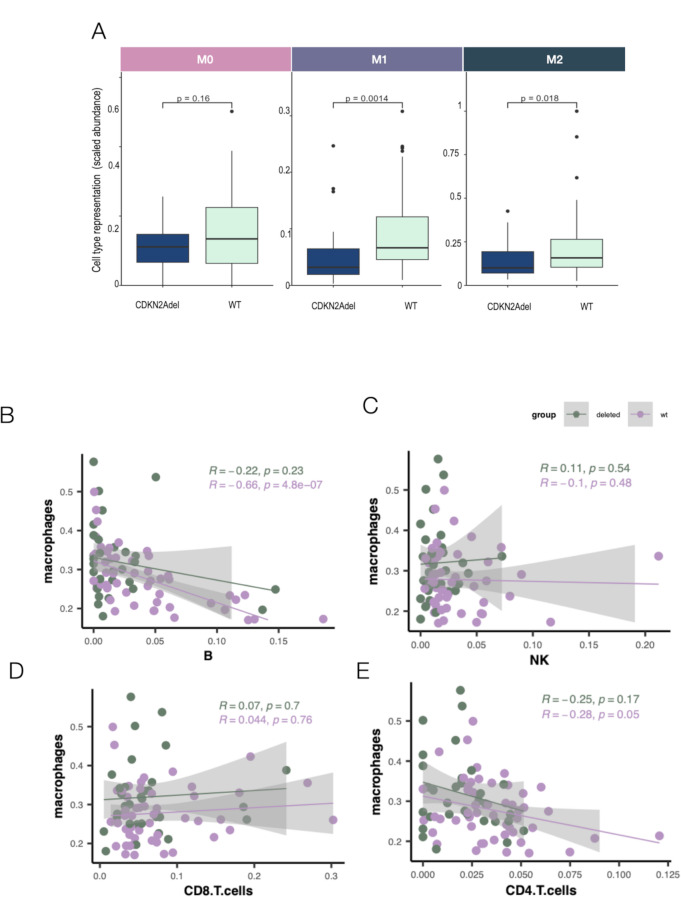
CDKN2Adel and macrophages in DPMs **A**. Deconvolution analysis in CDKN2Adel and WT DPMs of macrophage subpopulations. Boxplots represent the scaled abundance of the indicated macrophage subtypes estimated based on gene expression. **B-E**. Correlation plot of macrophages and B (**B**), NK (**C**), CD8 + T (**D**) and CD4 + T (**E**) cells predicted abundances in CDKN2Adel and WT DPMs. Spearman’s correlation was applied to calculate R coefficient and Pvalue.

Our data, indicated that CDKN2Adel is associated with a deep inhibition of macrophages. Macrophages play a complex role in mesothelioma from the immune defense against asbestos in the early phases of the disease, to a support to tumor growth, immune evasion, and resistance to therapy, associated with their persistent activation and subsequent polarization to an M2-like phenotype. To further investigate this aspect, we analyzed the impact of CDKN2Adel on the macrophage polarization in our samples. Using a deconvolution approach, we observed a significant decreased of both M1 and M2 cells in CDKN2Adel DPMs as compared to WT (Fig. 3A). Furthermore, the analysis of the relative distribution of these subpopulations did not reveal significant differences between WT and deleted samples, indicating that CDKN2Adel is associated with an overall loss of macrophages in tumor tissue rather than a perturbation of their functional state (data not shown). Macrophages are known to exert a significant function in shaping the asset of TiME. We explored the potential correlation between their predicted abundance and major immune effector cells (CD4 + and CD8 + T cells, B-cells and NKs) in both WT and CDKN2Adel DPM groups. Coherently with the reported pro-tumorigenic role of macrophages in DPMs we observed a significant negative correlation between macrophages and CD4 + T cells and B Cells in WT samples(Fig. 3B-E and Supplementary Fig. 7). Noticeably, in the CDKN2Adel these correlations were abolished (Fig. 3B-E).

#### Loss of chemotactic signaling impairs immune populations' recruitment in CDKN2Adel DPM and affects patients' survival

**Fig. 4:**
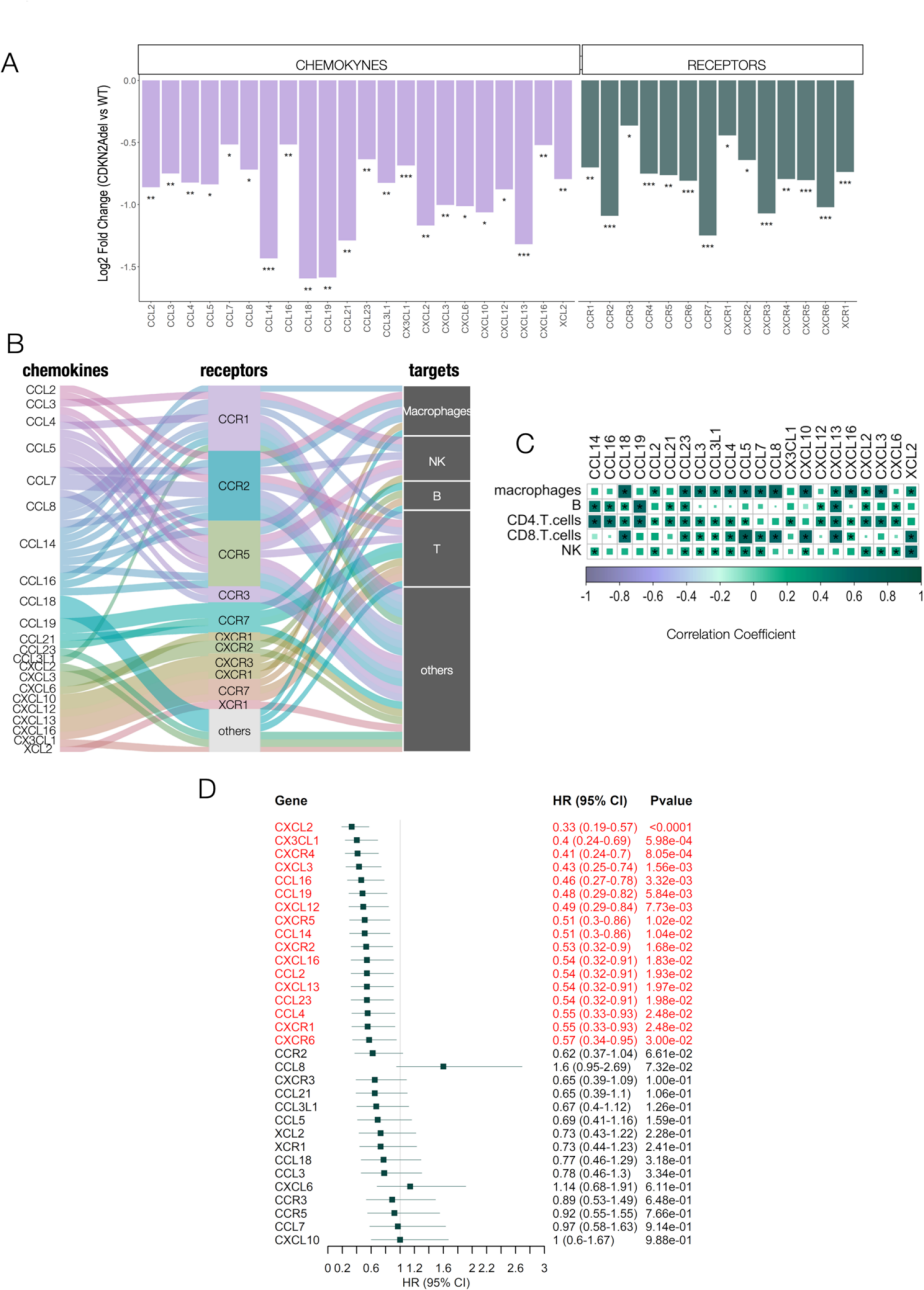
CDKN2Adel impairs chemotactic signals **A**. Bar plots report the differential expression of chemokines and related receptors significantly deregulated in gene expression analysis. For each gene the Y-axis represents the Log2Fold Change, in CDKN2Adel vs. WT DPMs. **B**. Alluvial plot showing the functional connection between deregulated cytokines, cytokine receptors and their target immune populations. Flows are colored according to cytokines. **C**. Correlation analysis between the expression of deregulated chemokines and the predicted abundance of the indicated immune populations. Asterisks indicate significant correlations (PValue < 0.05). Color intensity and square size are proportional to Spearman’s correlation coefficients. **D**. Forest plot illustrating the correlation between the expression of the indicated signaling genes and 2- years survival in our cohort. The cohort was dichotomized based on the median expression value of each gene. Green squares indicate hazard ratio value and horizontal lines represent 95% confidence

We then explored the relationship between the depletion of stimulatory signals and the configuration of the immune asset in CDKN2Adel DPMs. Several chemotactic cytokines and their associated receptors were strongly downregulated in CDKN2Adel as compared with WT DPMs. (Fig. 4A). Alluvial plot in Fig. 4B displays the chemokines and receptors that we found affected by CDKN2Adel in our analysis and the multilayered signaling axes that connect these molecules to their target immune populations. This prediction was validated in our cohort by a correlation analysis that showed a strong and widespread correlation between the expression of affected chemokines and the estimated cell abundance of effector cells in our samples (Fig. 4C). In particular, macrophages, immune scavengers, strongly correlated with most deregulated chemokines, whereas effector cells displayed selective correlations tied to their specific signaling [43].

Survival analysis demonstrated that the expression of several of these chemokines and receptors was significantly associated with an improved survival probability in our cohort (Fig. 4D) indicating that the perturbation of these molecules may account at least in part for the clinical aggressiveness associated with the CDKN2Adel.

#### Spatial transcriptomics shows that the immune-suppressive effect of CDKN2Adel is enriched in the sarcomatoid phenotype

Tumor infiltrating immune cells are major players in DPM clinical and histological progression and we recently reported that a dense immune infiltration is a distinctive feature of the sarcomatoid histotype versus the epithelioid one [20].

We employed a spatial transcriptomic approach to assess whether CDKN2Adel had a differential impact according to the histotype.

To this end, we analyzed transcriptional changes in the expression of 1800 immune-related genes, associated with CDKN2Adel specifically in epithelioid (E) and sarcomatoid (S) areas from biphasic or histologically pure DPMs.

**Fig. 5:**
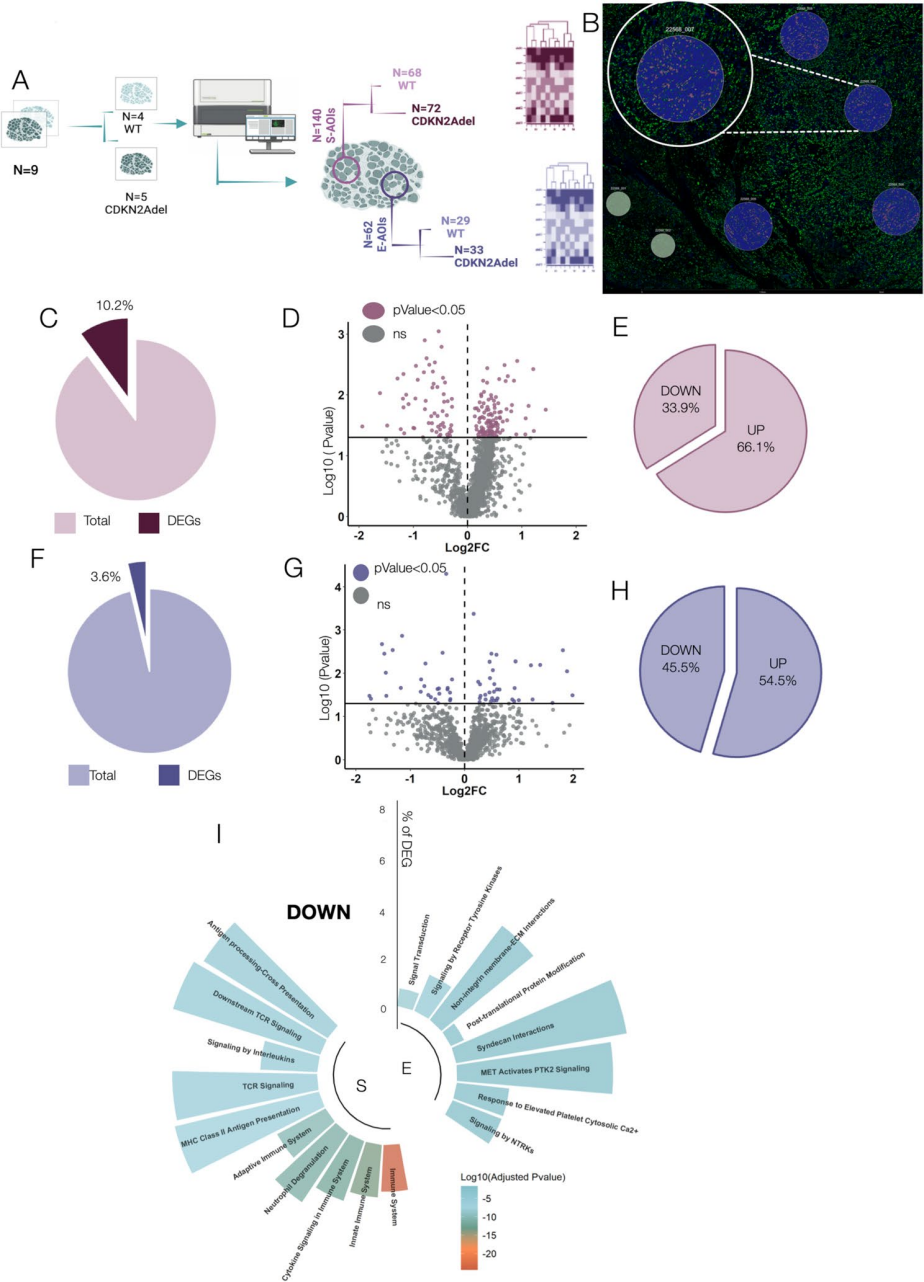
Differential effect of CDKN2Adel in epithelioid and sarcomatoid histotype **A**. Experimental design of the spatial transcriptomic analysis. Histological sections from 9 surgically resected DPMs were analyzed (N = 5 CDKN2adel, N = 4 WT). Epithelioid (E) and sarcomatoid (S) areas of illumination (AOIs) were selected based on cell morphology and expression of the Pan-CK marker. Collected AOIs were sequenced, and the expression of 1834 genes was analyzed. **B**. GeoMx DSP scan showing representative AOIs collected from a biph-DPM surgical biopsy. Insight displays the enlargement of representative transitional areas. Large circles identify selected biphasic areas. E-AOIs (red) and S-AOIs (blu) were selected based on morphology and Pan-CK signals. Small, light green circles indicate not segmented pure S-AOIs. **C**. Pie chart representing the percentage of DEGs (PValue < 0.05) in CDKN2Adel versus WT S-AOIs. **D**. Volcano plot showing expression and PValue of DEGs in the S-AOIs. Burgundy dots represent significantly deregulated genes (PValue < 0.05). **E**. Pie chart showing the distribution of downregulated and upregulated DEGs in S-AOIs. **F**. Pie chart representing the percentage of DEGs (PValue < 0.05) in CDKN2Adel versus WT E-AOIs. **G**. Volcano plot showing expression and PValue of DEGs in the E-AOIs. Violet dots represent significantly deregulated genes (Pvalue < 0.05). **H**. Pie chart shows the distribution of downregulated and upregulated DEGs in E-AOIs. **I**. GO analysis of downregulated genes in S-AOIs and E-AOIs. Circular histograms illustrate the top-scoring biological categories. Colors gradient represents significance (Log10 adjusted Pvalue) while bars height represents the percentage of involved DEGs for each category

5 CDKN2Adel and 4 WT DPM samples were analyzed. A total of 202 Areas Of Illumination (AOIs) were transcriptionally profiled, of which 140 Sarcomatoid-AOIs (S-AOIs) and 62 Epithelioid-AOIs (E-AOIs). Collectively, 97 AOIs belonged to WT DPM and 105 to CDKN2Adel DPMs (Fig. 5A-B and Supplementary Fig. 8).

Histotype-associated differential analysis identified 183 DEGs (10.2%) in CDKN2Adel as compared to WT in the S-AOIs. Of these, 121 (66.1%) were upregulated and 62 (33.8%) were downregulated (Fig. 5C-E) in mutated samples. Conversely, a markedly lower number of DEGs was observed in the E-AOIs. Only 66 genes (3.6%) were differentially expressed in the comparison of CDKN2Adel vs. WT group in the epithelioid histotype. 36 genes (54.5%) were upregulated and 30 (45.5%) downregulated in mutated samples (Fig. 5F-H).

Functional annotation showed that 79.4% of CDKN2Adel downregulated genes in the S-AOIs were involved in immunity-regulation and GO analysis confirmed the significant enrichment of processes involved in cytokine signaling and adaptive immunity, in line with the results obtained in the bulk transcriptomic analysis (Fig. 5I).

GO analysis of downregulated genes in the E-AOIs showed enrichment in signal transduction and cell-extracellular matrix interaction (Fig. 5I).

Interestingly, a greater degree of similarity between the two histotypes was observed for the biological pathways associated with upregulated genes. Several categories including gene expression, signal transduction RNA-PolII mediated transcription and SUMOylation were commonly affected in the two morphological compartments, further emphasizing the difference observed between the two histotypes for the immune-modulatory effect (Supplementary Fig. 9A).

**Fig. 6:**
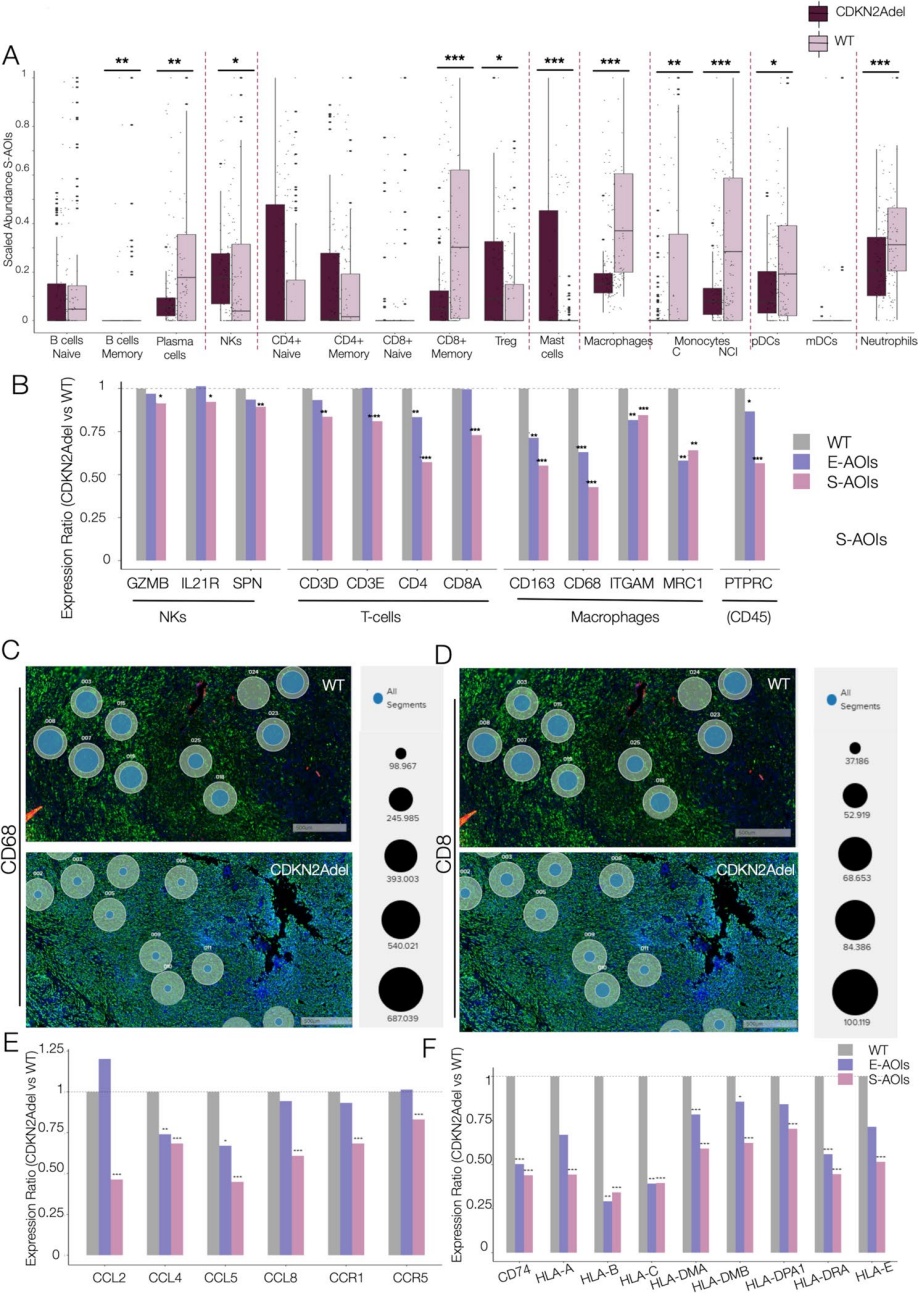
CDKN2Adel causes spatial tumor exclusion of immune cells in the S-AOIs **A**. Deconvolution analysis based on gene expression profile in S-AOIs. Bar plot shows the scaled abundance of each indicated population in CDKN2Adel and WT samples. **B**. Histograms showing the relative expression of lineage specific markers in CDKN2Adel as compared to WT DPMs (gray bars) in E-AOIs (purple bars) and S-AOIs (pink bars). **C-D**. Bubble plots showing expression of CD68 (C) and CD8 (D) in representative transitional areas, showing the difference between WT and CDKN2Adel S-AOIs. Large circles represent the selected AOIs. Blu dots inside the circle represent the level of expression of the indicated genes. Dots is proportional to the number of counts as reported in the figure. **E-F**. Histograms showing the relative expression of cytokines (E) and MHC genes (F) in E-AOIs (purple bars) and S-AOIs (pink bars) CDKN2Adel as compared to WT DPMs (grey bars). Pvalue: *0.01–0.05, ** 0.001–0.01 *** <0.001

Deconvolution analysis based on gene expression confirmed that CDKN2Adel causes a severe immune depletion in the S-AOIs (Fig. 6A).

The entire innate immunity compartment including macrophages, monocytes, dendritic cells, neutrophils and NKs as well as the B compartment (memory and plasma cells) showed severe depletion in the CDKN2Adel S-AOIs. In the T compartment, CD4 + T cells did not display significant alterations while CD8 + memory T cells were strongly depleted in the mutated areas. Of note, Mast Cells and T-regulatory cells (Tregs) showed an opposite trend being enriched in CDKN2Adel group as compared with WT group, only in the S-AOIs. Both cell types are implicated in anti-inflammatory and immune suppression processes. A coherent but more attenuated effect was observed in the E-AOIs were only a few cell populations displayed a significant inhibition in CDKN2Adel (Supplementary Fig. 9B). In particular, in these areas, the exclusion of effector cells associated with the CDKN2Adel appeared to be more significantly attenuated (for CD8 + T cells) or completely absent (B/NKs). These data were confirmed by the analysis of some immune markers (Fig. 6B). In particular, the cytotoxic T- lymphocyte marker CD8 was markedly decreased in CDKN2Adel S-AOIs but its expression was not significantly affected in E-AOIs. Conversely, abundance of macrophages was predicted as strongly impaired in both S- and E-AOIs (Fig. 6A and Supplementary Fig. 9B) and macrophage markers (CD68, MRC1 and ITGAM) were consistently downregulated in both S-AOIs and E-AOIs. Spatial breakdown of the transcriptomic data for CD68 (Fig. 6C) and CD8 (Fig. 6D) in CDKN2Adel and WT S-AOIs evidenced how their downregulation was not confined to specific areas but widely distributed across the entire tumor topography, confirming the systemic effect induced by the deletion (Supplementary Fig. 10).

In both the S- and E- compartments, we also confirmed the depletion of immunostimulatory signals including several chemotactic cytokines and MHC-I and II molecules, even if the extent of perturbation was greater in the S-AOIs (Fig. 6E-F).

## Discussion

**Fig. 7:**
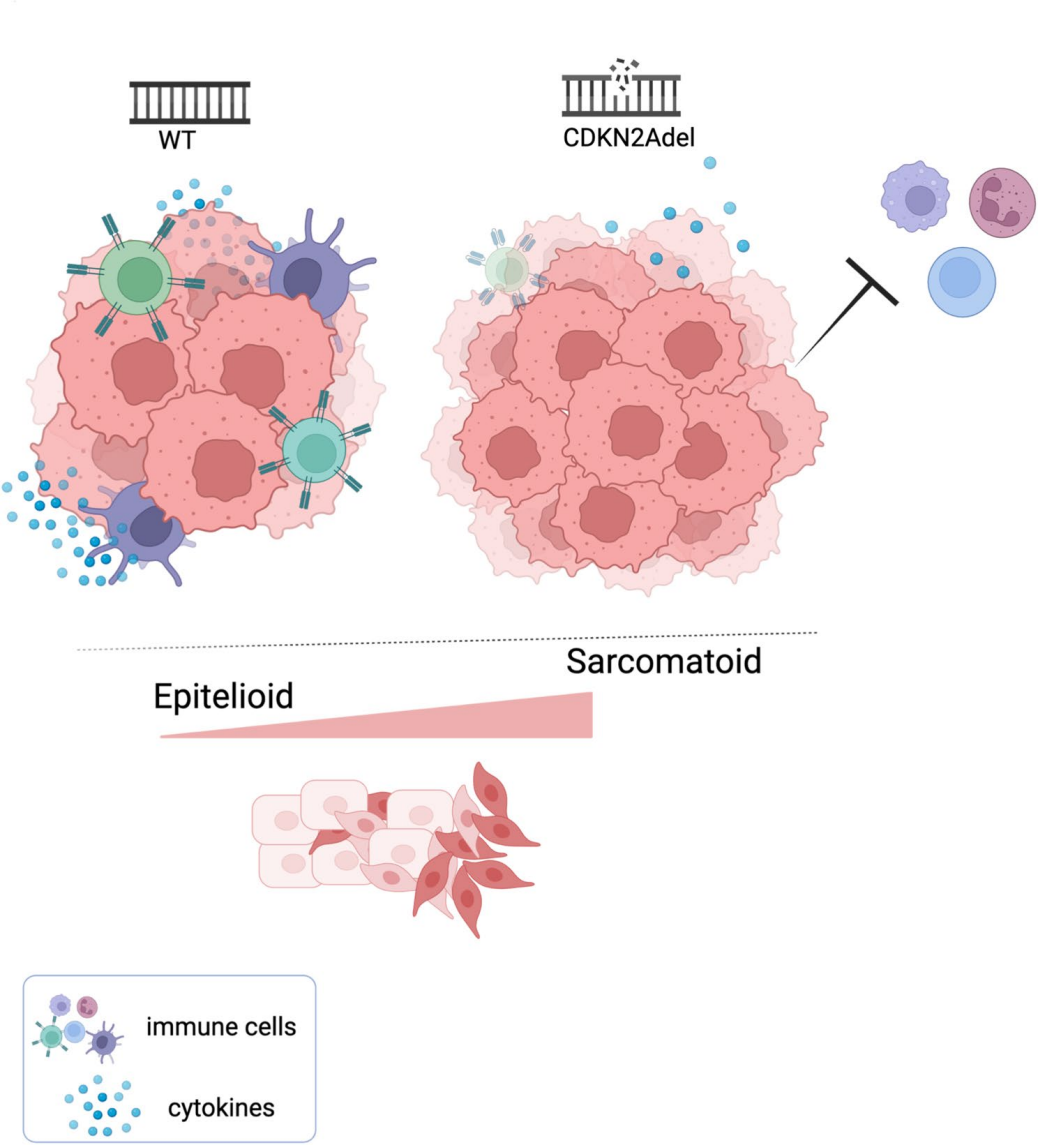
(Graphical Abstract) Schematic representation of the proposed model. In DPM CDKN2Adel is associated with a decrease of the immune-activating signals, in particular of chemotactic cytokines, resulting in the exclusion of infiltrating immune cells in the tumor milieu. This effect is preferentially associated with the Sarcomatoid phenotypes and likely contributes to fostering aggressiveness of these lesions

DPM is a current clinical emergency. Median overall survival is approximately one year, and treatment options are limited and poorly effective [1, 2]. The lack of prognostic biomarkers further complicates decision-making, while a complex intrinsic biology (that just recently began to emerge) delays the development of disease-oriented innovative approaches. In this study we explored the potential impact of CDKN2Adel on the organization and function of TiME in the context of DPM. Our data demonstrated that, this deletion is associated with a wide immune desert in which the tumor microenvironment appears depleted of immune infiltrate (Fig. 7). Main immune populations, both innate (NK, macrophages, monocytes etc.) and adaptive (B and T) cells, appeared to be affected and excluded from the tumor milieu in those lesions carrying the deletion.

These results are consistent with previous reports from other tumor settings [4, 8, 28–30]. Han et al. performed immune-genomic analysis from multiple TCGA datasets and demonstrated that 9p21 loss associates with a cold TiME in 10 out of 12 tumor types with frequent 9p21 loss, characterized by reduced abundance of B/T/NK cells, altered spatial TILs patterns and decreased expression of classical immune checkpoints (PD-L1) [29]. Similarly, Deng et al. and Gutionov et al. reported a significant immune depletion following CDKN2A deletion in gastric cancer and non-small cell lung cancer [30]. Besides, supporting these data, a potential correlation between deletion in the CDKN2A locus and a cold TiME had already emerged in the context of broad multiomics analysis in DPM [7, 8, 38, 44],. Indeed, our data extend this observation, providing a detailed characterization of the dynamics that underlie the immune-repressor effect of CDKN2Adel in this disease. We showed that chemotactic signaling is deeply perturbed in lesions harboring this genetic alteration. Main chemokines resulted significantly downregulated in CDKN2Adel DPM and their levels significantly associated with the estimated overall amount of effector cells carrying the matched receptors (B/T/NK cells). The clinical relevance of these observations was further supported by the fact that a significant number of CDKN2Adel deregulated chemokines displayed a protective effect on patients’ survival.

It is conceived that wide chromosomal alterations may stimulate immune activation by increasing the load of neoantigens that may trigger the activation of a cytotoxic immune response [21, 45]. However, other chromosomal deletions besides the one in the CDKN2A locus were previously reported to have an immunosuppressive phenotype in DPM [46]. For example, deletion of the 10q24.32 locus was reported to be associated with downregulation of prominent T-cell genes and with downregulated monocyte/macrophage and dendritic cell markers, suggesting a potential immune modulatory effect. Likely, the affection of CDKN2A adjacent genes within the 9p21.3 locus may concur to the “non-canonical effect” of this genetic alteration. Indeed, hot spot alterations in this region may affect up to 25 protein coding genes flanking the CDKN2A locus, including other cell cycle regulators (CDKN2B and KLHL9), a metabolic enzyme (MTAP), and a cluster of 16 IFN-I-related genes.

In particular, the IFN-I cluster has been proven to be relevant in the immune-modulatory effect associated with the 9p21.3 genetic deletion in cancer. In lung cancer, patients with co-occurring CDKN2A, CDKN2B and IFN-I cluster deletion display shorter survival as compared with the ones carrying only deletion of CDKN2A [47]. In a syngenic mouse model of pancreatic cancer co-deletion of the IFN-I cluster promoted immune evasion and immunotherapy resistance. In this model, the destruction of IFN-signaling caused marked changes in infiltrating immune cells and the insurgence of escape mechanisms from CD8 + T-cell surveillance [48]. Intriguingly, our data envisage either a marginal involvement of IFN I cluster co-deletion or the activation of compensatory mechanisms in the context of DPM. Indeed, we observed that co-deletion of IFN I cluster is associated with a significant perturbation of the IFN I signaling pathway (Supplementary Fig. 3) but not with a marked effect on the immune phenotype associated with CDKN2Adel (Fig. 1C and Supplementary Fig. 5). Even if potentially affected by the limited sample size and by the detection method used, we believe that these results are indicative of the fact that the causes of the immune desertification observed are likely attributable to other determinants. In this regard, it is interesting to note that also, the deletion of MTAP, a key metabolic enzyme involved in the methionine salvage pathway, which is co-deleted with CDKN2A and B in up to 90% of cases, has been associated with immunosuppressive profile in cancer and with alternative macrophage polarization [49]. Future studies employing functional models for the analysis of the interaction dynamics between tumor cells (CDKN2Adel vs. WT) and immune cells should be implemented to clarify how this genetic alteration reshapes DPM microenvironment to foster immune evasion. Among the elements of novelty introduced by this work there is the use of a spatial transcriptomics approach to dissect the impact of CDKN2Adel according to the histotype.

By this approach, we discovered that the immunosuppression, associated with CDKN2Adel, has a differential extension and it is more marked in the sarcomatoids histotype (Fig. 6). The preferential effect of CDKN2Adel in the sarcomatoid phenotype may just reflect the presence of a denser immune infiltrate associated with the sarcomatoid component [3, 20], or vice versa being one of the biological mechanisms with which this genetic lesion supports the aggressiveness and progression of DPM. In this regard, we were very much intrigued by the observation that, while effector cells of both adaptive (B and T lymphocytes) and innate immunity (NK) were almost exclusively impaired in the sarcomatoid histotype, macrophages and their precursors, monocytes, were profoundly affected in both compartments. This observation seemed to indicate that the block of monocytes-macrophages chemotaxis could be a primary consequence of CDKN2Adel, in line with the current state of the art that identifies these cells and their functional heterogeneity as an integral part of the microenvironmental contribution to the evolution of DPM [3, 4, 20]. Coherent with this hypothesis, we observed that CDKN2Adel selectively abolishes the correlation between macrophages and the immune effector populations (Fig. 3). This further highlights the complex role of macrophages in DPM and confirms that the inhibition of these cells may be pivotal for the immunosuppressed phenotype associated with CDKN2Adel. Furthermore, it is important to note that the presence of the deletion, while causing a significant decrease in macrophage abundance, does not alter their polarization process, further supporting the hypothesis that it is a blockage of their chemotaxis and not an alteration of their function that is impaired in DPMs with cDKN2Adel. The strong decrease observed in the expression of chemotactic cytokines would be consistent with this model.

## Conclusion

In conclusion, our data show that in DPM, the deletion of CDKN2A is associated with a significant depletion of stimulatory and chemotactic signals, resulting in the exclusion of immune infiltrate from tumor parenchyma. This immune desertification effect is prominent in the sarcomatoid lesions and likely contributes to the clinical aggressiveness associated with CDKN2Adel. These results may be translationally relevant for the use of immunotherapy in DPMs. Indeed, while ICIs defined an improved outcomes for a subset of DPM patients, the identification of reliable predictive biomarkers for response remains a significant challenge. In this regard, many biomarkers including PD-L1 expression, Tumor Mutational Burden (TMB) and DPMs frequent genetic alterations (like BAP1, TP53 and NF2) were explored but none reached a sufficient performance [11, 15, 16, 50–52], Currently, histology stands as the most important determinant for ICIs efficacy. For this reason, a new frontier of the field could be implementing combinations of molecular biomarkers with histology for selecting patients to undergo immunotherapy in DPMs. Our results provide a strong rational to test CDKN2Adel together with histology as predictive criteria for immunotherapy in DPM, as already suggested in other settings [28–30, 53]. Indeed, it is plausible that sarcomatoid DPMs harboring CDKN2Adel respond less effectively to immunotherapy than WT sarcomatoid DPMs, due to the immune desertification effect associated with this deletion.

## Supplementary Information

Below is the link to the electronic supplementary material.

Supplementary Material 1

## Data Availability

Digital Barcoding dataset is available at GEO accession number: GSE302048. Spatial Transcriptomic data are available at ArrayExpress accession number: E-MTAB-15378.

## Abbreviations

DPM: Diffuse Pleural Mesothelioma
ICIs: Immune checkpoint inhibitors
CDKN2Adel: CDKN2A deleted
IFN Idel: IFN I cluster deletion
WT: CDKN2A wild type
TiME: Tumor immune microenvironment
FFPE: Formalin-Fixed Paraffin-Embedded
FC: Fold Change
E: Epithelioid
S: Sarcomatoid
Biph: Biphasic
ROI: Region of interest
NGS: Next Generation Sequencing
QC: Quality Check
SD: Standard Deviation
Q3: Third quartile
DEGs: Differentially expressed genes
GO: Gene Ontology
AOI: Area of Illumination
Tregs: T-regulatory cells
DSP: Digital Spatial Profiler
ddPCR: digital droplets PCR

## References

[1] Janes SM, Alrifai D, Fennell DA. Perspectives on the treatment of malignant pleural mesothelioma. N Engl J Med. 2021;385:1207–18. 34551230 10.1056/NEJMra1912719

[2] Robinson BM. Malignant pleural mesothelioma: an epidemiological perspective. Ann Cardiothorac Surg. 2012;1:491–6. 23977542 10.3978/j.issn.2225-319X.2012.11.04 PMC3741803

[3] Giotti B, Dolasia K, Zhao W, Cai P, Sweeney R, Merritt E, Kiner E, Kim GS, Bhagwat A, Nguyen T, et al. Single-Cell view of tumor microenvironment gradients in pleural mesothelioma. Cancer Discov. 2024;14:2262–78. 38959428 10.1158/2159-8290.CD-23-0017 PMC13109001

[4] Mangiante L, Alcala N, Sexton-Oates A, Di Genova A, Gonzalez-Perez A, Khandekar A, Bergstrom EN, Kim J, Liu X, Blazquez-Encinas R, et al. Multiomic analysis of malignant pleural mesothelioma identifies molecular axes and specialized tumor profiles driving intertumor heterogeneity. Nat Genet. 2023;55:607–18. 36928603 10.1038/s41588-023-01321-1 PMC10101853

[5] Galateau Salle F, Le Stang N, Tirode F, Courtiol P, Nicholson AG, Tsao MS, Tazelaar HD, Churg A, Dacic S, Roggli V, et al. Comprehensive molecular and pathologic evaluation of transitional mesothelioma assisted by deep learning approach: A Multi-Institutional study of the international mesothelioma panel from the MESOPATH reference center. J Thorac Oncol. 2020;15:1037–53. 32165206 10.1016/j.jtho.2020.01.025 PMC8864581

[6] Blum Y, Meiller C, Quetel L, Elarouci N, Ayadi M, Tashtanbaeva D, Armenoult L, Montagne F, Tranchant R, Renier A, et al. Dissecting heterogeneity in malignant pleural mesothelioma through histo-molecular gradients for clinical applications. Nat Commun. 2019;10:1333. 30902996 10.1038/s41467-019-09307-6 PMC6430832

[7] Bueno R, Stawiski EW, Goldstein LD, Durinck S, De Rienzo A, Modrusan Z, Gnad F, Nguyen TT, Jaiswal BS, Chirieac LR, et al. Comprehensive genomic analysis of malignant pleural mesothelioma identifies recurrent mutations, gene fusions and splicing alterations. Nat Genet. 2016;48:407–16. 26928227 10.1038/ng.3520

[8] Hmeljak J, Sanchez-Vega F, Hoadley KA, Shih J, Stewart C, Heiman D, Tarpey P, Danilova L, Drill E, Gibb EA, et al. Integrative molecular characterization of malignant pleural mesothelioma. Cancer Discov. 2018;8:1548–65. 30322867 10.1158/2159-8290.CD-18-0804 PMC6310008

[9] Fennell DA, Dulloo S, Harber J. Immunotherapy approaches for malignant pleural mesothelioma. Nat Rev Clin Oncol. 2022;19:573–84. 35778611 10.1038/s41571-022-00649-7

[10] Peters S, Scherpereel A, Cornelissen R, Oulkhouir Y, Greillier L, Kaplan MA, Talbot T, Monnet I, Hiret S, Baas P, et al. First-line nivolumab plus ipilimumab versus chemotherapy in patients with unresectable malignant pleural mesothelioma: 3-year outcomes from checkmate 743. Ann Oncol. 2022;33:488–99. 35124183 10.1016/j.annonc.2022.01.074

[11] Quispel-Janssen J, van der Noort V, de Vries JF, Zimmerman M, Lalezari F, Thunnissen E, Monkhorst K, Schouten R, Schunselaar L, Disselhorst M, et al. Programmed death 1 Blockade with nivolumab in patients with recurrent malignant pleural mesothelioma. J Thorac Oncol. 2018;13:1569–76. 29908324 10.1016/j.jtho.2018.05.038

[12] Baas P, Scherpereel A, Nowak AK, Fujimoto N, Peters S, Tsao AS, Mansfield AS, Popat S, Jahan T, Antonia S, et al. First-line nivolumab plus ipilimumab in unresectable malignant pleural mesothelioma (CheckMate 743): a multicentre, randomised, open-label, phase 3 trial. Lancet. 2021;397:375–86. 33485464 10.1016/S0140-6736(20)32714-8

[13] Meirson T, Pentimalli F, Cerza F, Baglio G, Gray SG, Correale P, Krstic-Demonacos M, Markel G, Giordano A, Bomze D, Mutti L. Comparison of 3 randomized clinical trials of frontline therapies for malignant pleural mesothelioma. JAMA Netw Open. 2022;5:e221490. 35262715 10.1001/jamanetworkopen.2022.1490 PMC8908075

[14] Nakamura A, Hashimoto M, Kondo N, Matsumoto S, Kuroda A, Minami T, Kitajima K, Kuribayashi K, Kijima T, Hasegawa S. Efficacy and safety of nivolumab with ipilimumab for recurrent malignant pleural mesothelioma after primary surgical intervention. Int J Clin Oncol. 2023;28:409–15. 36609928 10.1007/s10147-023-02292-3

[15] Perrino M, De Vincenzo F, Cordua N, Borea F, Aliprandi M, Santoro A, Zucali PA. Immunotherapy with immune checkpoint inhibitors and predictive biomarkers in malignant mesothelioma: work still in progress. Front Immunol. 2023;14:1121557. 36776840 10.3389/fimmu.2023.1121557 PMC9911663

[16] Marabelle A, Fakih M, Lopez J, Shah M, Shapira-Frommer R, Nakagawa K, Chung HC, Kindler HL, Lopez-Martin JA, Miller WH Jr., et al. Association of tumour mutational burden with outcomes in patients with advanced solid tumours treated with pembrolizumab: prospective biomarker analysis of the multicohort, open-label, phase 2 KEYNOTE-158 study. Lancet Oncol. 2020;21:1353–65. 32919526 10.1016/S1470-2045(20)30445-9

[17] Lorenzini E, Ciarrocchi A, Torricelli F. Molecular fingerprints of malignant pleural mesothelioma: not just a matter of genetic alterations. J Clin Med 2021, 10. 10.3390/jcm10112470 PMC8199660 34199544

[18] Pinato DJ, Mauri FA, Ramakrishnan R, Wahab L, Lloyd T, Sharma R. Inflammation-based prognostic indices in malignant pleural mesothelioma. J Thorac Oncol. 2012;7:587–94. 22307011 10.1097/JTO.0b013e31823f45c1

[19] Yang H, Rivera Z, Jube S, Nasu M, Bertino P, Goparaju C, Franzoso G, Lotze MT, Krausz T, Pass HI, et al. Programmed necrosis induced by asbestos in human mesothelial cells causes high-mobility group box 1 protein release and resultant inflammation. Proc Natl Acad Sci U S A. 2010;107:12611–6. 20616036 10.1073/pnas.1006542107 PMC2906549

[20] Torricelli F, Donati B, Reggiani F, Manicardi V, Piana S, Valli R, Lococo F, Ciarrocchi A. Spatially resolved, high-dimensional transcriptomics sorts out the evolution of biphasic malignant pleural mesothelioma: new paradigms for immunotherapy. Mol Cancer. 2023;22:114. 37460925 10.1186/s12943-023-01816-9 PMC10351128

[21] Mansfield AS, Peikert T, Smadbeck JB, Udell JBM, Garcia-Rivera E, Elsbernd L, Erskine CL, Van Keulen VP, Kosari F, Murphy SJ, et al. Neoantigenic potential of complex chromosomal rearrangements in mesothelioma. J Thorac Oncol. 2019;14:276–87. 30316012 10.1016/j.jtho.2018.10.001 PMC6348045

[22] Martinez-Jimenez F, Priestley P, Shale C, Baber J, Rozemuller E, Cuppen E. Genetic immune escape landscape in primary and metastatic cancer. Nat Genet. 2023;55:820–31. 37165135 10.1038/s41588-023-01367-1 PMC10181939

[23] Hoyos D, Zappasodi R, Schulze I, Sethna Z, de Andrade KC, Bajorin DF, Bandlamudi C, Callahan MK, Funt SA, Hadrup SR, et al. Fundamental immune-oncogenicity trade-offs define driver mutation fitness. Nature. 2022;606:172–9. 35545680 10.1038/s41586-022-04696-z PMC9159948

[24] Mizuno S, Yamaguchi R, Hasegawa T, Hayashi S, Fujita M, Zhang F, Koh Y, Lee SY, Yoon SS, Shimizu E, et al. Immunogenomic pan-cancer landscape reveals immune escape mechanisms and immunoediting histories. Sci Rep. 2021;11:15713. 34344966 10.1038/s41598-021-95287-x PMC8333422

[25] Vandenhoeck J, van Meerbeeck JP, Fransen E, Raskin J, Van Camp G, Op de Beeck K, Lamote K. DNA methylation as a diagnostic biomarker for malignant mesothelioma: A systematic review and Meta-Analysis. J Thorac Oncol. 2021;16:1461–78. 34082107 10.1016/j.jtho.2021.05.015

[26] Mavrakis KJ, McDonald ER 3rd, Schlabach MR, Billy E, Hoffman GR, deWeck A, Ruddy DA, Venkatesan K, Yu J, McAllister G, et al. Disordered methionine metabolism in MTAP/CDKN2A-deleted cancers leads to dependence on PRMT5. Science. 2016;351:1208–13. 26912361 10.1126/science.aad5944

[27] Minami JK, Morrow D, Bayley NA, Fernandez EG, Salinas JJ, Tse C, Zhu H, Su B, Plawat R, Jones A, et al. CDKN2A deletion remodels lipid metabolism to prime glioblastoma for ferroptosis. Cancer Cell. 2023;41:1048–e10601049. 37236196 10.1016/j.ccell.2023.05.001 PMC10330677

[28] Gutiontov SI, Turchan WT, Spurr LF, Rouhani SJ, Chervin CS, Steinhardt G, Lager AM, Wanjari P, Malik R, Connell PP, et al. CDKN2A loss-of-function predicts immunotherapy resistance in non-small cell lung cancer. Sci Rep. 2021;11:20059. 34625620 10.1038/s41598-021-99524-1 PMC8501138

[29] Han G, Yang G, Hao D, Lu Y, Thein K, Simpson BS, Chen J, Sun R, Alhalabi O, Wang R, et al. 9p21 loss confers a cold tumor immune microenvironment and primary resistance to immune checkpoint therapy. Nat Commun. 2021;12:5606. 34556668 10.1038/s41467-021-25894-9 PMC8460828

[30] Deng C, Li ZX, Xie CJ, Zhang QL, Hu BS, Wang MD, Mei J, Yang C, Zhong Z, Wang KW. Pan-cancer analysis of CDKN2A alterations identifies a subset of gastric cancer with a cold tumor immune microenvironment. Hum Genomics. 2024;18:55. 38822443 10.1186/s40246-024-00615-7 PMC11143690

[31] Yu J, Yan J, Guo Q, Chi Z, Tang B, Zheng B, Yu J, Yin T, Cheng Z, Wu X, et al. Genetic aberrations in the CDK4 pathway are associated with innate resistance to PD-1 Blockade in Chinese patients with Non-Cutaneous melanoma. Clin Cancer Res. 2019;25:6511–23. 31375512 10.1158/1078-0432.CCR-19-0475

[32] Chen Z, Guo Y, Zhao D, Zou Q, Yu F, Zhang L, Xu L. Comprehensive analysis revealed that CDKN2A is a biomarker for immune infiltrates in multiple cancers. Front Cell Dev Biol. 2021;9:808208. 35004697 10.3389/fcell.2021.808208 PMC8733648

[33] Liu S, Knochelmann HM, Lomeli SH, Hong A, Richardson M, Yang Z, Lim RJ, Wang Y, Dumitras C, Krysan K, et al. Response and recurrence correlates in individuals treated with neoadjuvant anti-PD-1 therapy for resectable oral cavity squamous cell carcinoma. Cell Rep Med. 2021;2:100411. 34755131 10.1016/j.xcrm.2021.100411 PMC8561238

[34] Luo JP, Wang J, Huang JH. CDKN2A is a prognostic biomarker and correlated with immune infiltrates in hepatocellular carcinoma. Biosci Rep 2021, 41. 10.1042/BSR20211103 PMC8495430 34405225

[35] Zhao L, Zhou X, Li H, Yin T, Jiang Y. Prognosis of immunotherapy for non-small cell lung cancer with CDKN2A loss of function. J Thorac Dis. 2024;16:507–15. 38410565 10.21037/jtd-23-1017 PMC10894420

[36] Banchereau R, Leng N, Zill O, Sokol E, Liu G, Pavlick D, Maund S, Liu LF, Kadel E 3rd, Baldwin N, et al. Molecular determinants of response to PD-L1 Blockade across tumor types. Nat Commun. 2021;12:3969. 34172722 10.1038/s41467-021-24112-w PMC8233428

[37] Xue L, Tang W, Zhou J, Xue J, Li Q, Ge X, Lin F, Zhao W, Guo Y. Next-generation sequencing identifies CDKN2A alterations as prognostic biomarkers in recurrent or metastatic head and neck squamous cell carcinoma predominantly receiving immune checkpoint inhibitors. Front Oncol. 2023;13:1276009. 37936609 10.3389/fonc.2023.1276009 PMC10627168

[38] Jang HJ, Truong CY, Lo EM, Holmes HM, Ramos D, Ramineni M, Lee JS, Wang DY, Pietropaolo M, Ripley RT, et al. Inhibition of Cyclin dependent kinase 4/6 overcomes primary resistance to programmed cell death 1 Blockade in malignant mesothelioma. Ann Thorac Surg. 2022;114:1842–52. 34592265 10.1016/j.athoracsur.2021.08.054 PMC8957629

[39] Manzotti G, Torricelli F, Benedetta D, Lococo F, Sancisi V, Rossi G, Piana S, Ciarrocchi A. An Epithelial-to-Mesenchymal transcriptional switch triggers evolution of pulmonary sarcomatoid carcinoma (PSC) and identifies dasatinib as new therapeutic option. Clin Cancer Res. 2019;25:2348–60. 30587547 10.1158/1078-0432.CCR-18-2364

[40] Luminari S, Donati B, Casali M, Valli R, Santi R, Puccini B, Kovalchuk S, Ruffini A, Fama A, Berti V, et al. A gene Expression-based model to predict metabolic response after two courses of ABVD in hodgkin lymphoma patients. Clin Cancer Res. 2020;26:373–83. 31645353 10.1158/1078-0432.CCR-19-2356

[41] Donati B, Reggiani F, Torricelli F, Santandrea G, Rossi T, Bisagni A, Gasparini E, Neri A, Cortesi L, Ferrari G, et al. Spatial distribution of immune cells drives resistance to neoadjuvant chemotherapy in Triple-Negative breast cancer. Cancer Immunol Res. 2024;12:120–34. 37856875 10.1158/2326-6066.CIR-23-0076

[42] Danaher P, Kim Y, Nelson B, Griswold M, Yang Z, Piazza E, Beechem JM. Advances in mixed cell Deconvolution enable quantification of cell types in Spatial transcriptomic data. Nat Commun. 2022;13:385. 35046414 10.1038/s41467-022-28020-5 PMC8770643

[43] Sokol CL, Luster AD. The chemokine system in innate immunity. Cold Spring Harb Perspect Biol 2015, 7. 10.1101/cshperspect.a016303 PMC4448619 25635046

[44] Alay A, Cordero D, Hijazo-Pechero S, Aliagas E, Lopez-Doriga A, Marin R, Palmero R, Llatjos R, Escobar I, Ramos R et al. Integrative transcriptome analysis of malignant pleural mesothelioma reveals a clinically relevant immune-based classification. J Immunother Cancer 2021, 9. 10.1136/jitc-2020-001601 PMC7908918 33632900

[45] Shi Y, Jing B, Xi R. Comprehensive analysis of neoantigens derived from structural variation across whole genomes from 2528 tumors. Genome Biol. 2023;24:169. 37461029 10.1186/s13059-023-03005-9 PMC10351168

[46] Nastase A, Mandal A, Lu SK, Anbunathan H, Morris-Rosendahl D, Zhang YZ, Sun XM, Gennatas S, Rintoul RC, Edwards M, et al. Integrated genomics point to immune vulnerabilities in pleural mesothelioma. Sci Rep. 2021;11:19138. 34580349 10.1038/s41598-021-98414-w PMC8476593

[47] Peng Y, Chen Y, Song M, Zhang X, Li P, Yu X, Huang Y, Zhang N, Ji L, Xia L, et al. Co-occurrence of CDKN2A/B and IFN-I homozygous deletions correlates with an immunosuppressive phenotype and poor prognosis in lung adenocarcinoma. Mol Oncol. 2022;16:1746–60. 35253368 10.1002/1878-0261.13206 PMC9019898

[48] Barriga FM, Tsanov KM, Ho YJ, Sohail N, Zhang A, Baslan T, Wuest AN, Del Priore I, Meskauskaite B, Livshits G, et al. MACHETE identifies interferon-encompassing chromosome 9p21.3 deletions as mediators of immune evasion and metastasis. Nat Cancer. 2022;3:1367–85. 36344707 10.1038/s43018-022-00443-5 PMC9701143

[49] Hansen LJ, Yang R, Roso K, Wang W, Chen L, Yang Q, Pirozzi CJ, He Y. MTAP loss correlates with an immunosuppressive profile in GBM and its substrate MTA stimulates alternative macrophage polarization. Sci Rep. 2022;12:4183. 35264604 10.1038/s41598-022-07697-0 PMC8907307

[50] Disselhorst MJ, Lubeck Y, van der Noort V, Quispel-Janssen J, Seignette IM, Sanders J, Peters D, Hooijberg E, Baas P. Immune cells in mesothelioma microenvironment simplistic marker of response to nivolumab plus ipilimumab? Lung Cancer. 2022;173:49–52. 36122471 10.1016/j.lungcan.2022.08.019

[51] Calabro L, Rossi G, Morra A, Rosati C, Cutaia O, Daffina MG, Altomonte M, Di Giacomo AM, Casula M, Fazio C, et al. Tremelimumab plus durvalumab retreatment and 4-year outcomes in patients with mesothelioma: a follow-up of the open label, non-randomised, phase 2 NIBIT-MESO-1 study. Lancet Respir Med. 2021;9:969–76. 33844995 10.1016/S2213-2600(21)00043-6 PMC9765708

[52] Zhang M, Bzura A, Baitei EY, Zhou Z, Spicer JB, Poile C, Rogel J, Branson A, King A, Barber S, et al. A gut microbiota rheostat forecasts responsiveness to PD-L1 and VEGF Blockade in mesothelioma. Nat Commun. 2024;15:7187. 39168966 10.1038/s41467-024-49842-5 PMC11339264

[53] Adib E, Nassar AH, Akl EW, Abou Alaiwi S, Nuzzo PV, Mouhieddine TH, Sonpavde G, Haddad RI, Mouw KW, Giannakis M, et al. CDKN2A alterations and response to immunotherapy in solid tumors. Clin Cancer Res. 2021;27:4025–35. 34074656 10.1158/1078-0432.CCR-21-0575 PMC8900067

