# Supplementary material for: CDKN2A deletion is associated with immune desertification in diffuse pleural mesothelioma
Source: J Exp Clin Cancer Res. 2025 Aug 28;44:256. doi: 10.1186/s13046-025-03522-4 (PMC12392524; doi:10.1186/s13046-025-03522-4)
Supplement: Supplementary file 1 — Supplementary Material 1 [file 13046_2025_3522_MOESM1_ESM.pdf]

Supplementary Figure 1

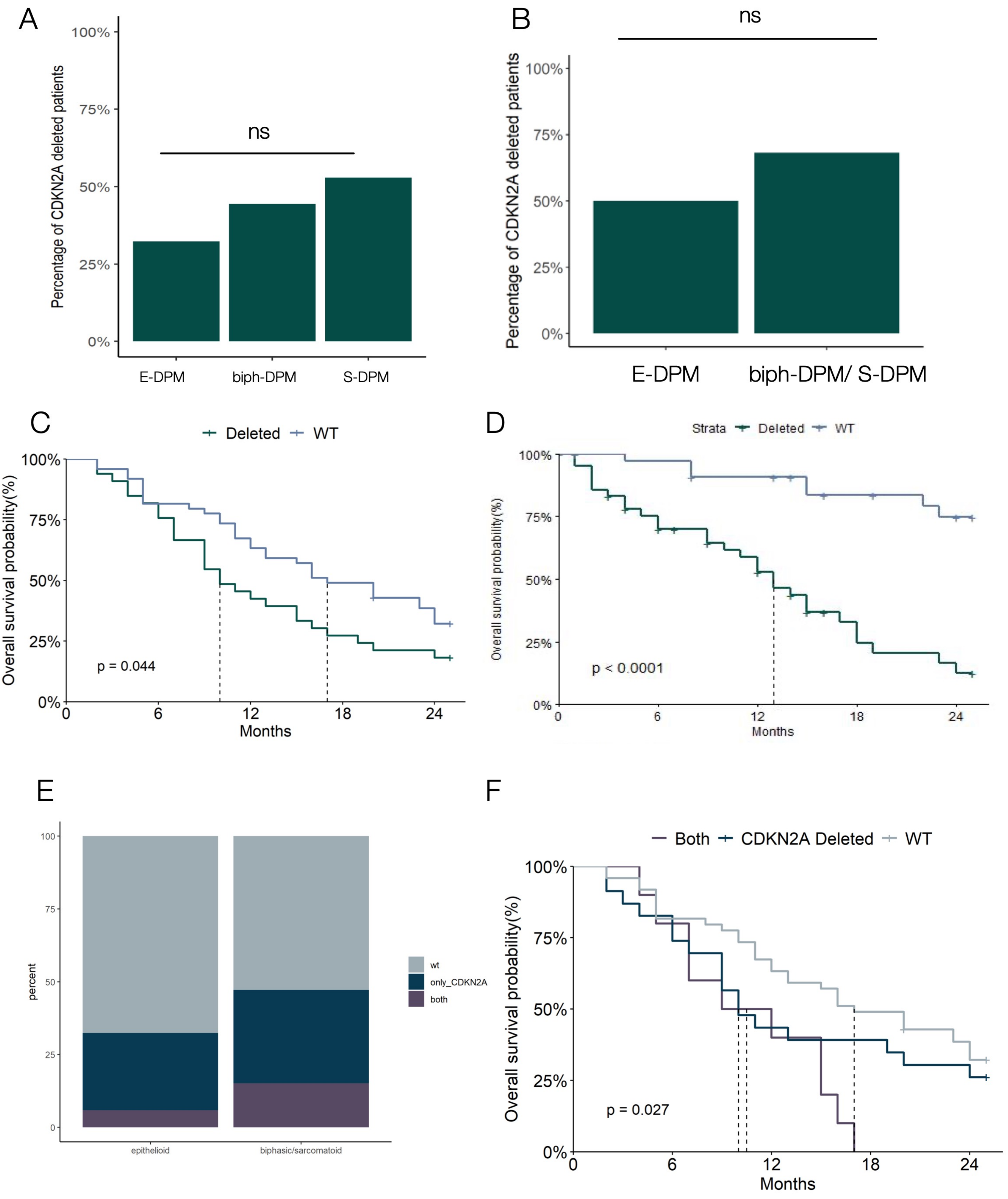

Supplementary Figure 2

A

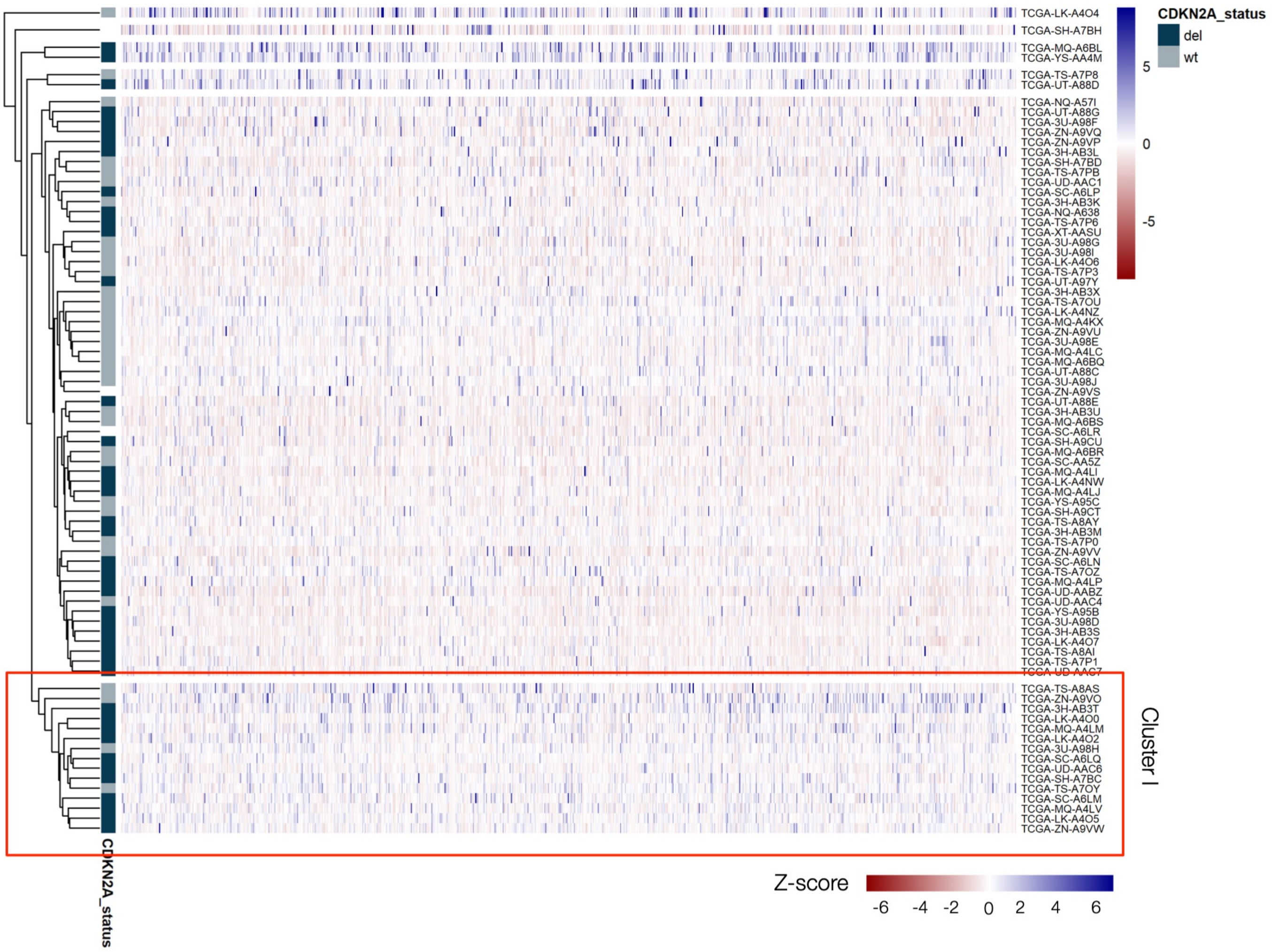

Supplementary Figure 3

A

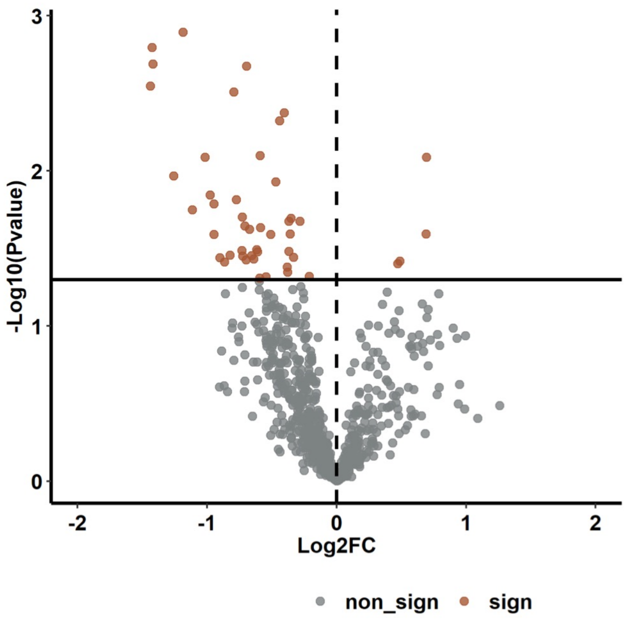

B

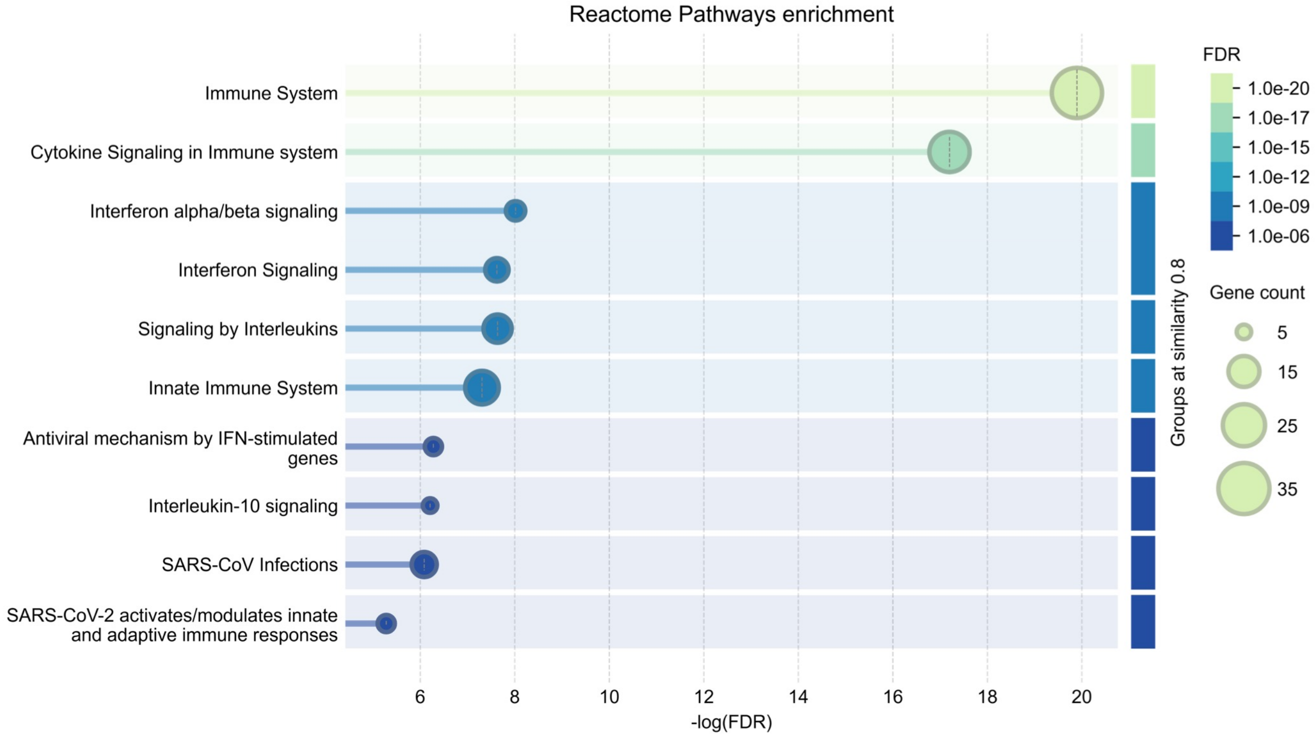

Supplementary Figure 4

A

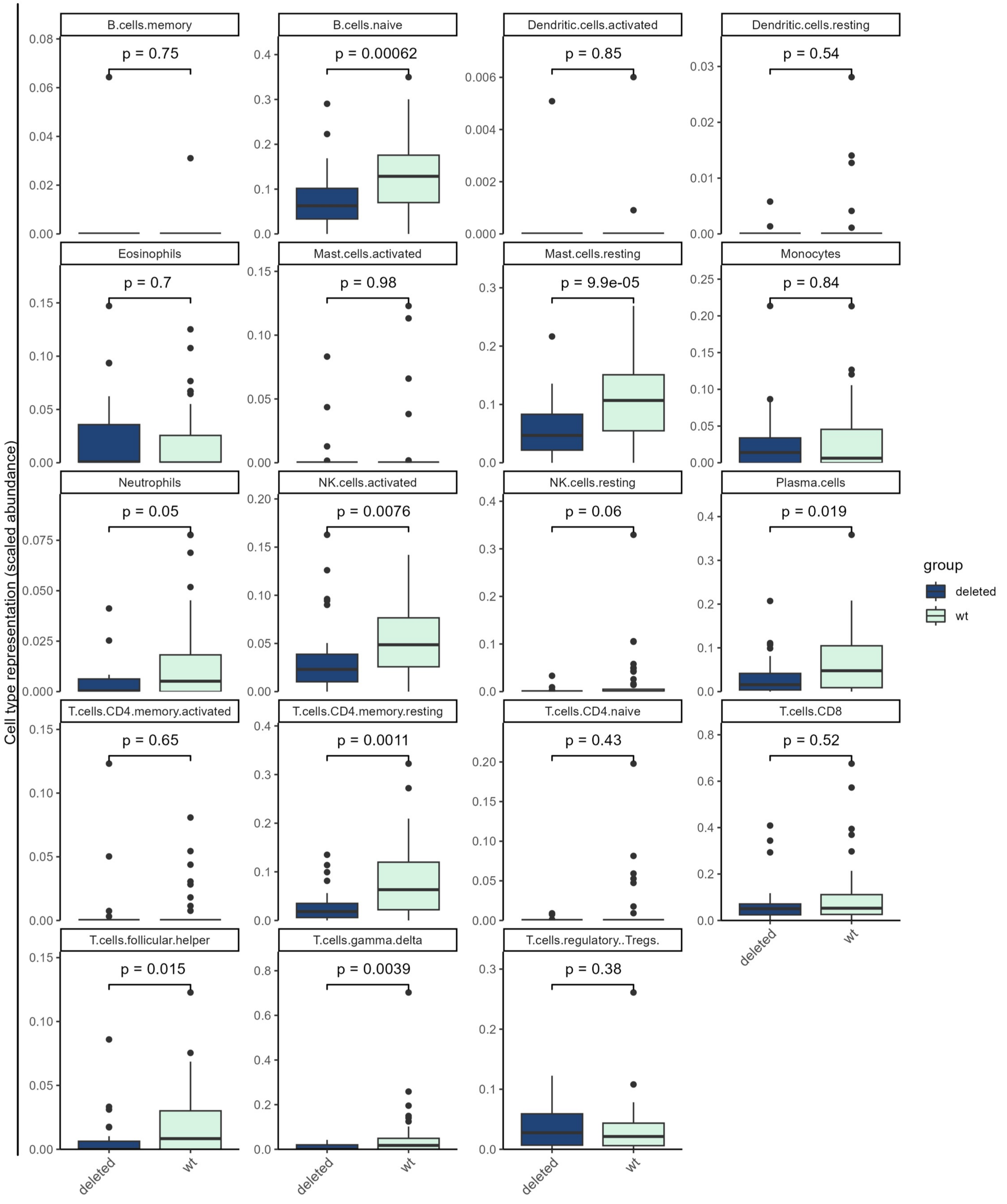

Supplementary Figure 5

A

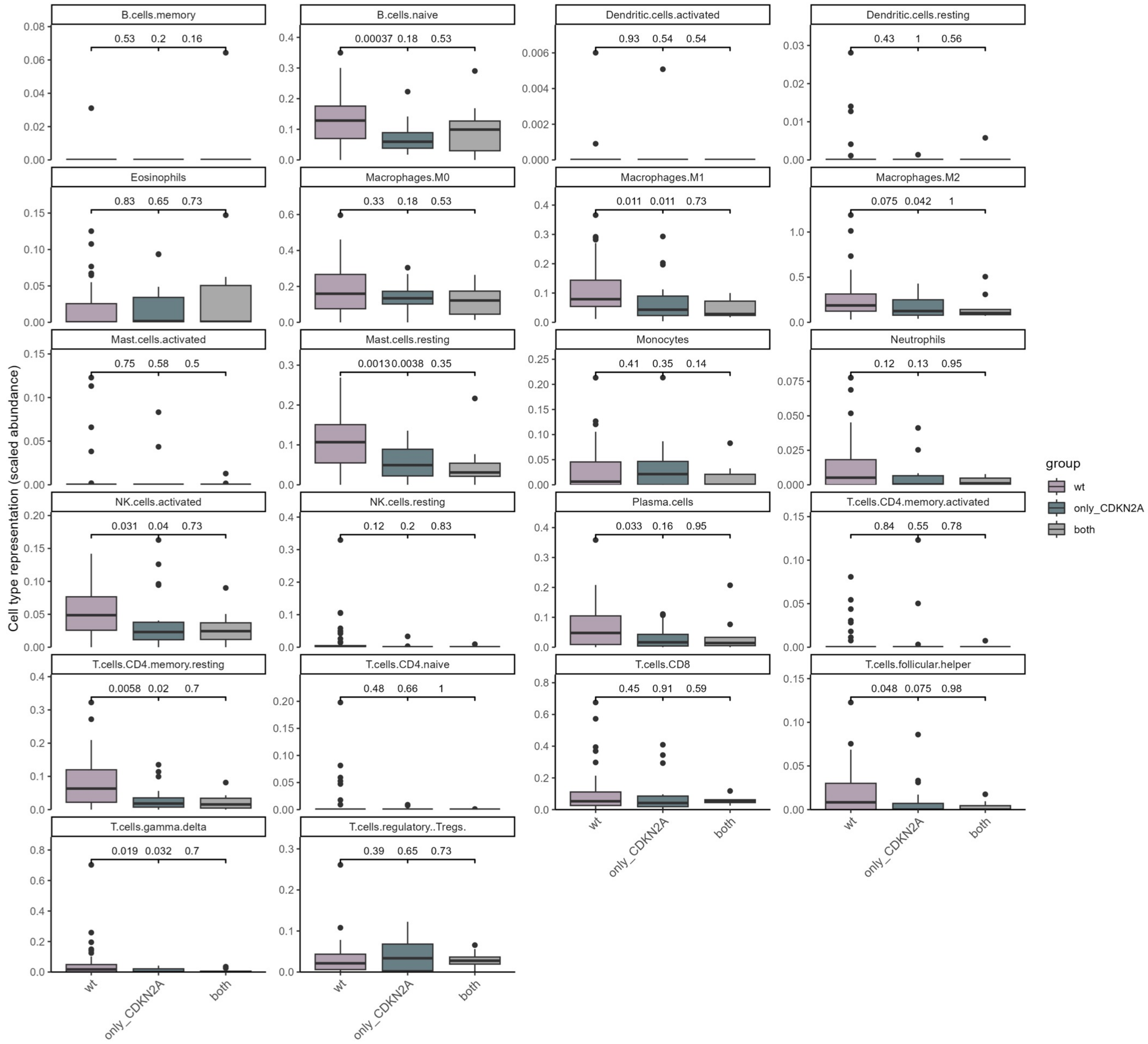

Supplementary Figure 6

A

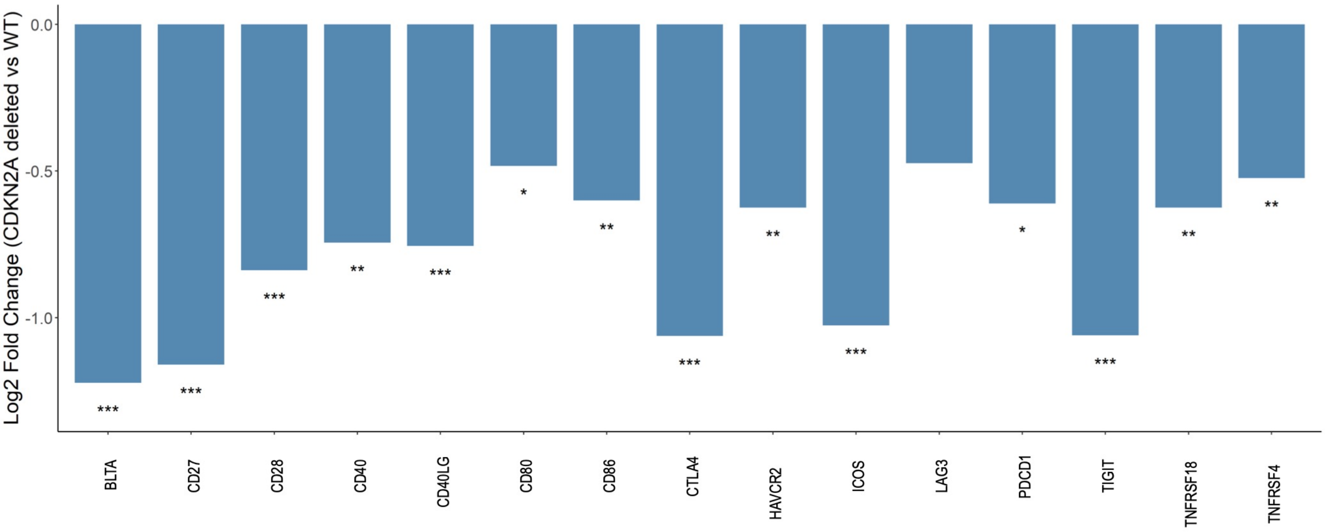

Supplementary Figure 7

A

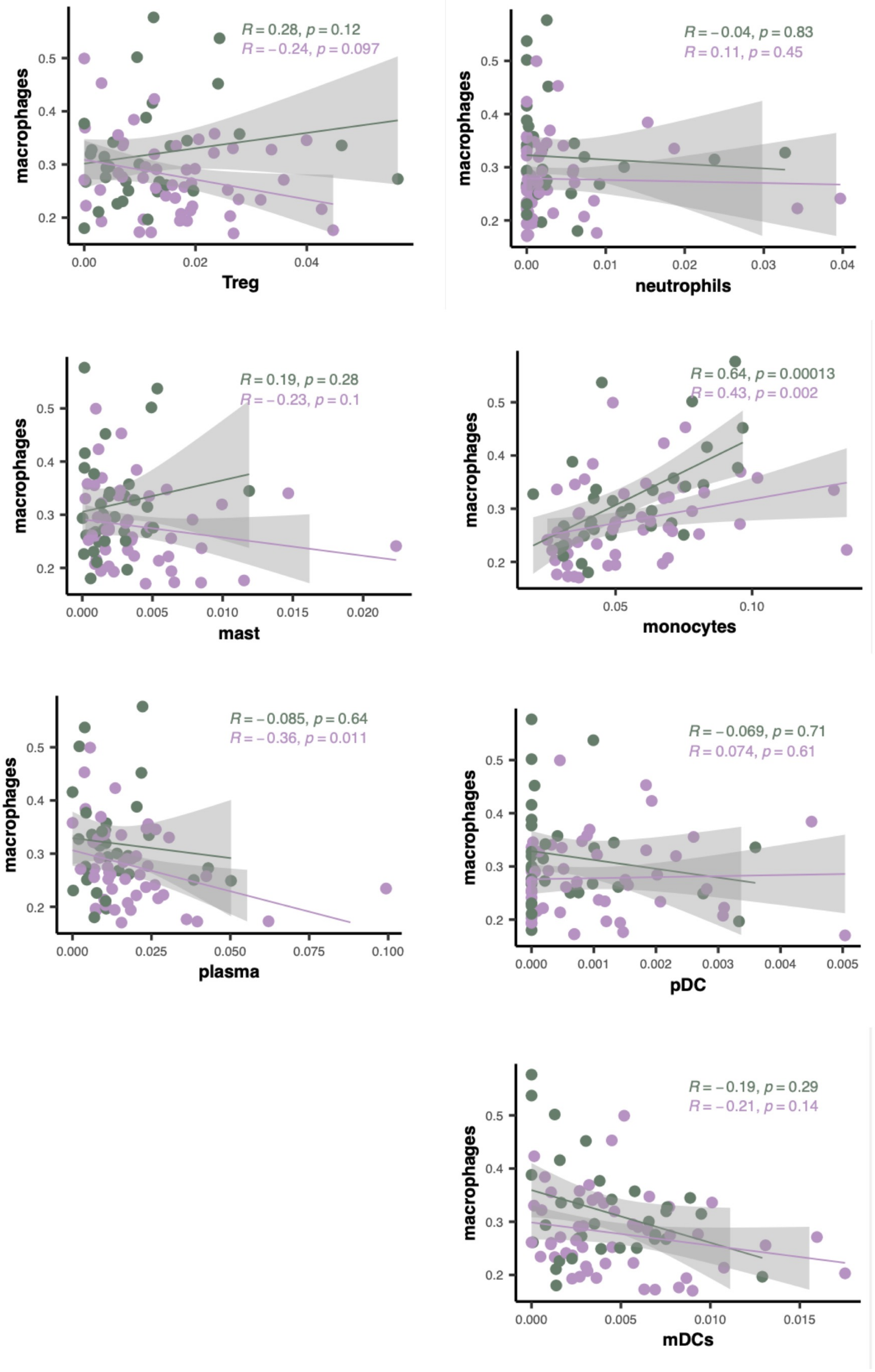

Supplementary Figure 8

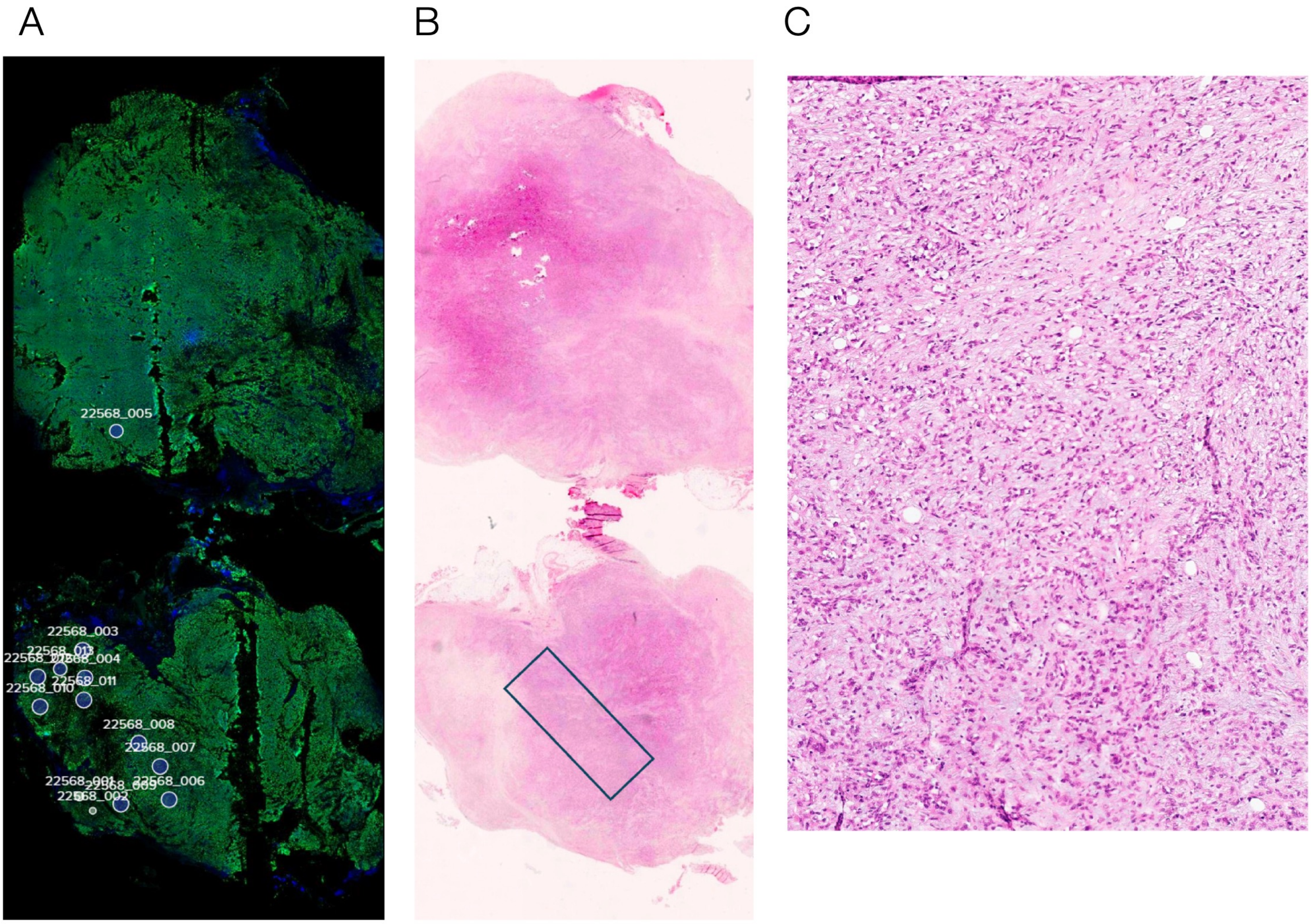

Full images relative to Figure 5B

Supplementary Figure 9

A

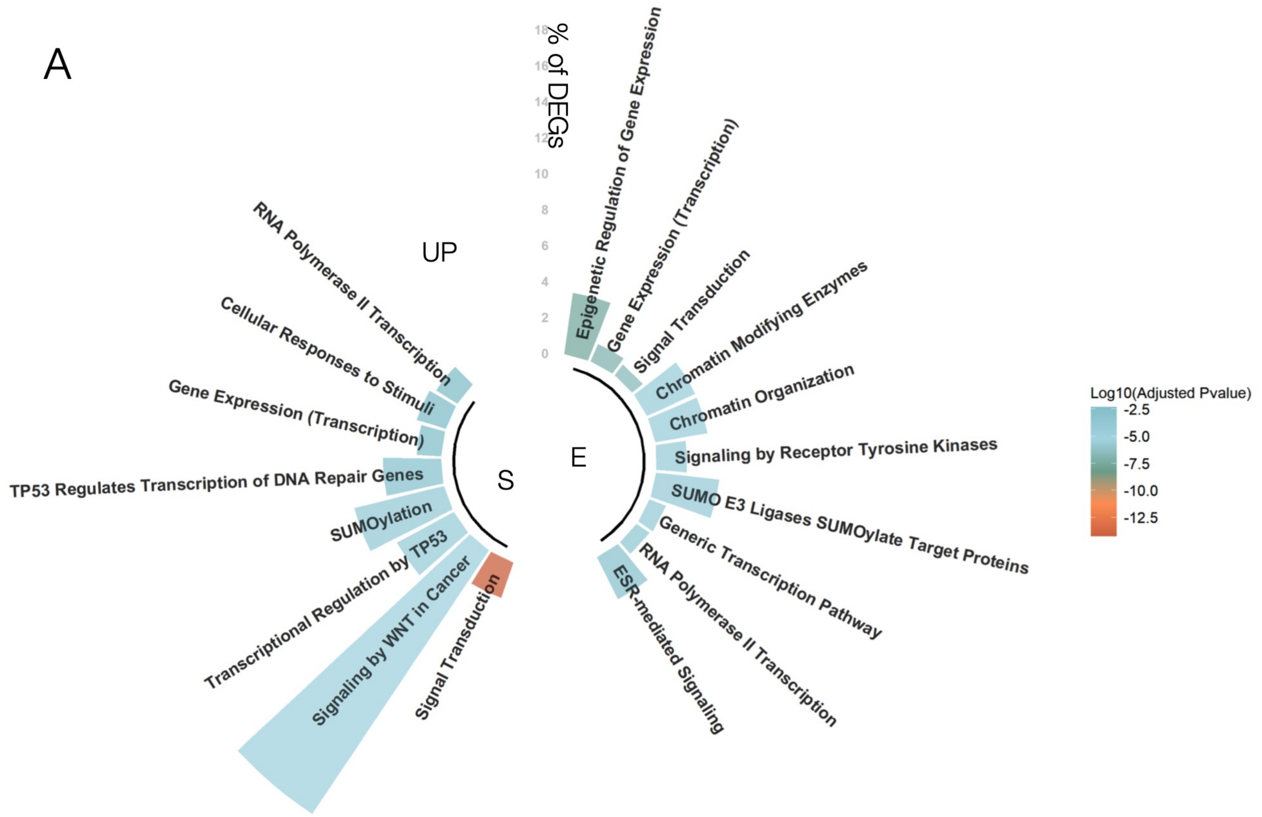

B

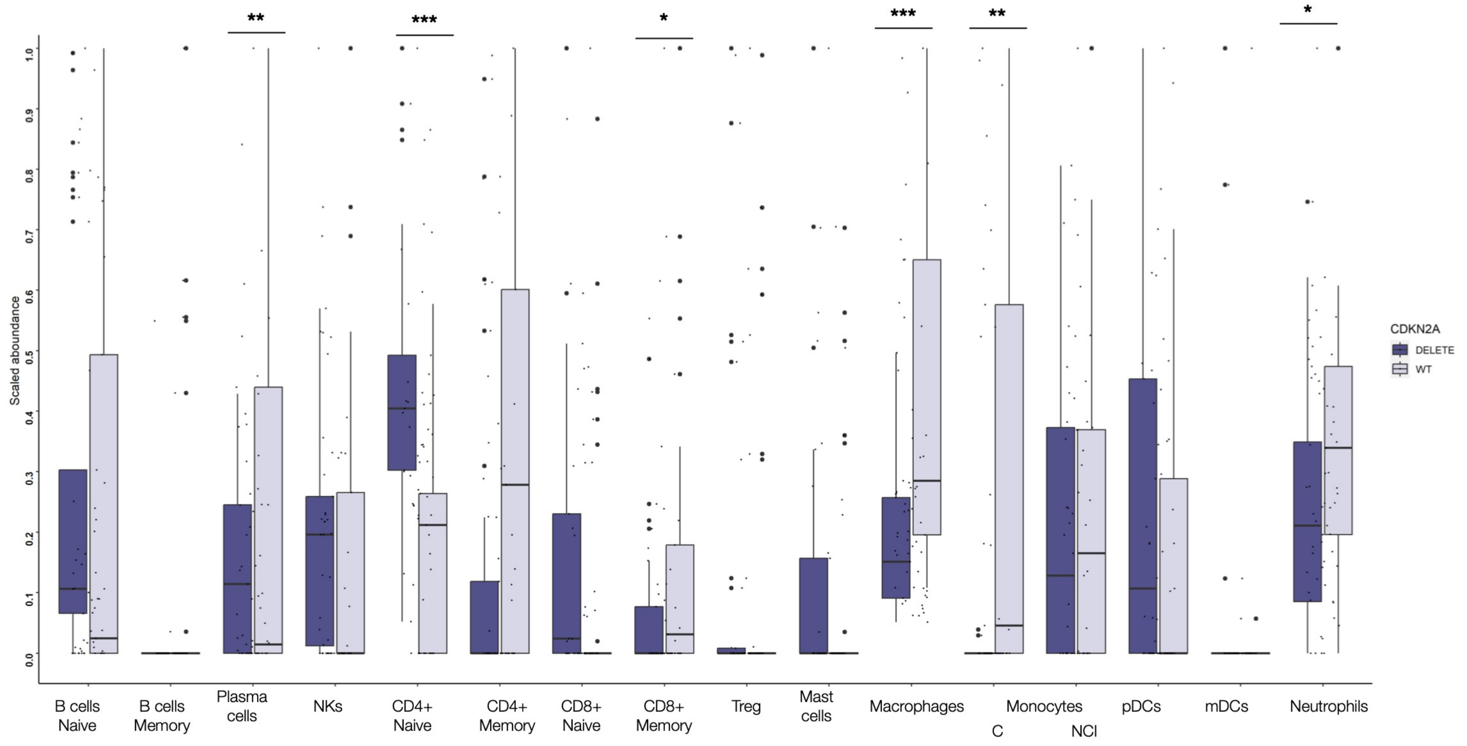

Supplementary Figure 10

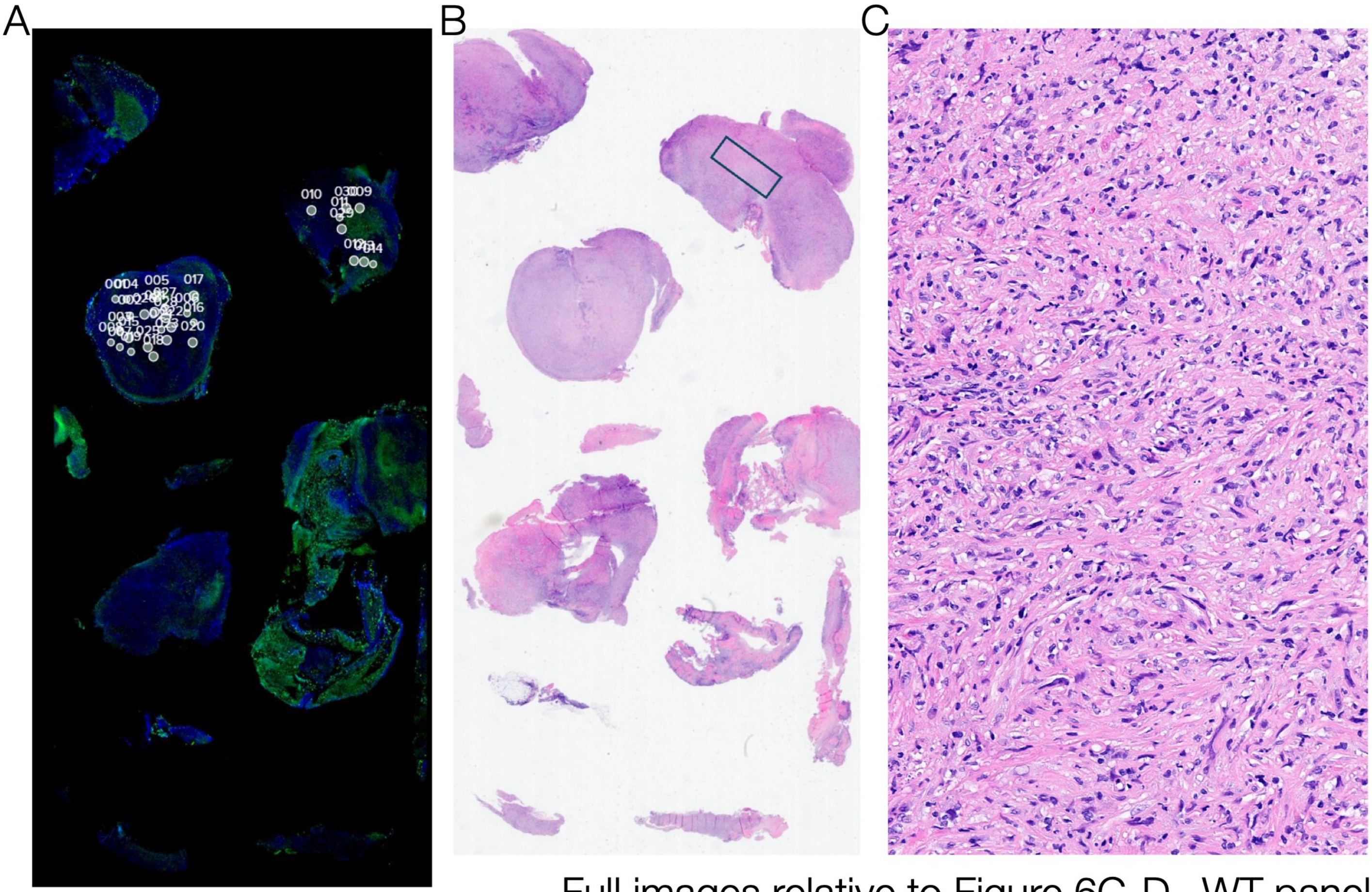

Full images relative to Figure 6C-D_ WT panel

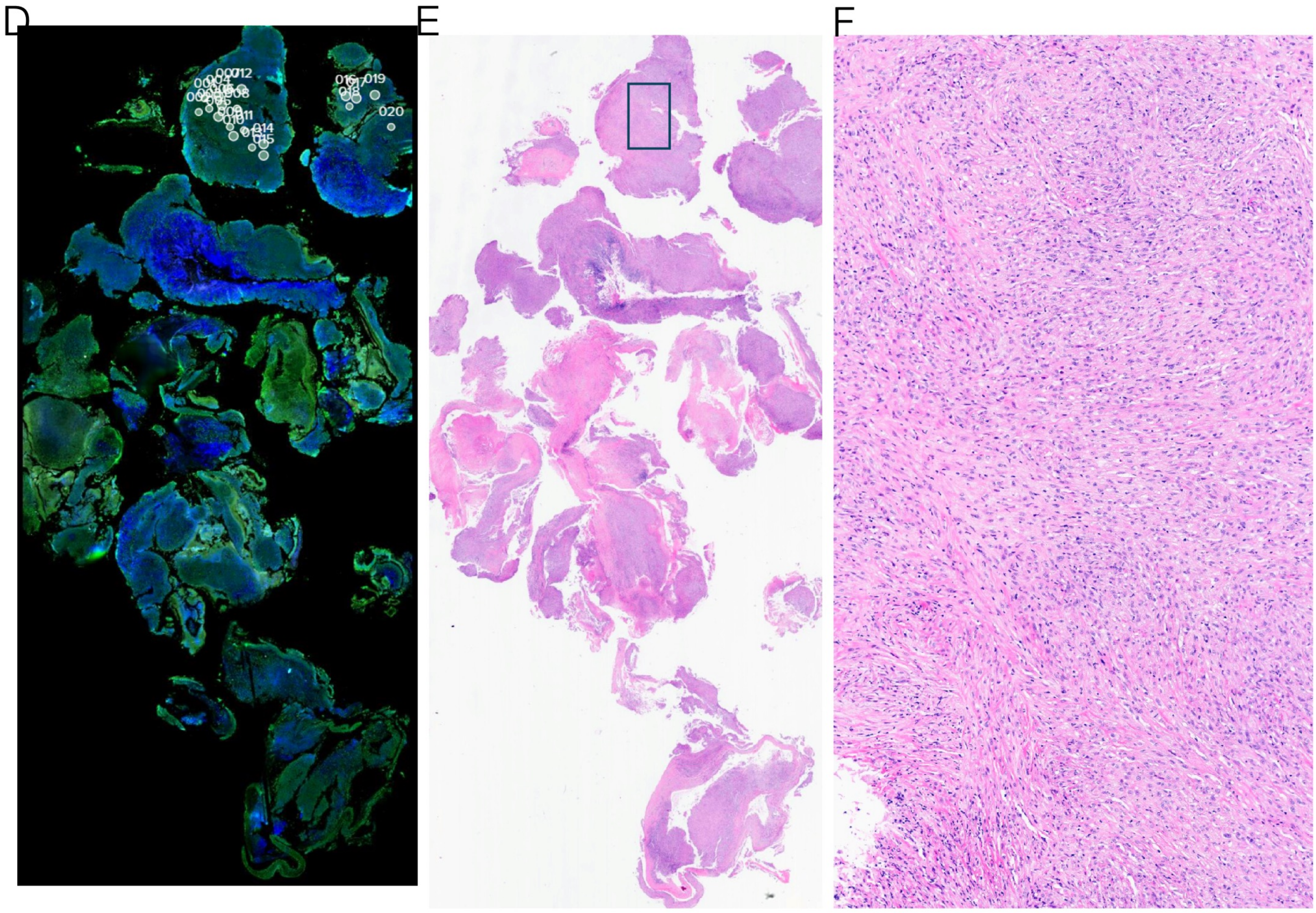

Full images relative to Figure 6C-D_ CDKN2Adel panel

Supplementary Table I

	DELETED (N=37)	WT (N=52)	Total (N=89)	p value
**Sex**				0.283
F	5 (27.8%)	13 (72.2%)	18 (100.0%)	
M	30 (44.1%)	38 (55.9%)	68 (100.0%)	
N-Miss	2	1	3	
**Age**				0.810
Mean (SD)	70.5 (8.8)	70.9 (8.4)	70.7 (8.5)	
N-Miss	1	1	2	
**Side**				0.894
left	12 (40.0%)	18 (60.0%)	30 (100.0%)	
na	0 (0.0%)	1 (100.0%)	1 (100.0%)	
right	24 (44.4%)	30 (55.6%)	54 (100.0%)	
N-Miss	1	3	4	
**Histology**				0.334
biphasic	16 (44.4%)	20 (55.6%)	36 (100.0%)	
epithelioid	11 (32.4%)	23 (67.6%)	34 (100.0%)	
sarcomatoid	9 (52.9%)	8 (47.1%)	17 (100.0%)	
N-Miss	1	1	2	
**Stage**				0.237
I	18 (46.2%)	21 (53.8%)	39 (100.0%)	
II	0 (0.0%)	4 (100.0%)	4 (100.0%)	
III	4 (57.1%)	3 (42.9%)	7 (100.0%)	
IV	12 (37.5%)	20 (62.5%)	32 (100.0%)	
N-Miss	3	4	7	

Supplementary figure Legends

Supplementary Figure S1

A. Bar plot representing the percentage of CDKN2A deleted patients subdivided by histotype in our cohort of 89 DPMs. B. Bar plot representing the percentage of CDKN2A deleted patients subdivided by histotype in the TCGA-MESO cohort (N=74). C. Kaplan Meier curves of CDKN2Adel and WT DPMs in our cohort (N=89). D. Kaplan Meier curves of CDKN2Adel and WT DPMs in the TCGA-MESO cohort (N=74). E. Bar plot showing the distribution of WT, CDKN2Adel and CDKN2Adel/IFN Idel (both), subdivided by histotype. F. Kaplan Meier curves of CDKN2Adel/IFN Idel (both), CDKN2Adel and WT DPMs in our cohort (N=89).

Supplementary Figure S2

C. Unsupervised clustering analysis based on the 770 genes of the ncounter immune-profile panel on the TCGA-MESO cohort. Bar plots reporting the differential expression of immune-checkpoints . For each gene the Y-axis represents the Log2FC, in CDKN2Adel vs WT MPMs. P-values: *0.01-0.05, ** 0.001-0.01 *** <0.001

Supplementary Figure S3

A.Volcano plot showing the expression and Pvalue of DEGs in CDKN2Adel/IFN Idel versus CDKN2Adel DPMs B.Lollipop plot showing principal Reactome pathways significatively deregulated in CDKN2Adel/IFN Idel versus CDKN2Adel DPMs (https://string-db.org/)

Supplementary Figure S4

Box plots showing deconvolution results obtained from validation analysis performed by CIBERSORTx comparing CDKN2Adel and WT nCounter data.

Supplementary Figure S5

Box plots showing deconvolution results obtained from validation analysis performed by CIBERSORTx comparing CDKN2Adel/IFN I del, CDKN2Adel and WT nCounter data.

Supplementary Figure S6

Bar plots reporting the differential expression of immune-checkpoints . For each gene the Y-axis represents the Log2FC, in CDKN2Adel vs WT DPMs. P-values: *0.01-0.05, ** 0.001-0.01 *** <0.001

Supplementary Figure S7

Correlation plot of macrophages and other immune populations predicted abundances in CDKN2Adel and WT DPMs. Spearman's correlation was applied to calculate R coefficient and Pvalue.

Supplementary Figure S8

A.Whole-slide GeoMx Digital Spatial Profiler (DSP) scan, B. Hematoxylin and eosin (H&E) staining C. Magnified view of the H&E-stained section of the area in which spatial transcriptomics was performed for the representative WT DPM displayed in Figure 5B. Green: PanCK, Blue: SYTO-13 nuclear staining.

Supplementary Figure S9

A. GO analysis of upregulated genes in S-AOIs and E-AOIs. Circular histograms illustrate the top-scoring biological categories. Colors gradient represents significance (Log10 adjusted pValue) while bars height represents the percentage of involved DEGs for each category. B. Deconvolution analysis based on gene expression profile in E-AOIs. Bar plot shows the scaled abundance of each indicated population in CDKN2Adel and WT samples. P-values: *0.01-0.05, ** 0.001-0.01 *** <0.001.

Supplementary Figure S10

A.Whole-slide GeoMx Digital Spatial Profiler (DSP) scan, B. Hematoxylin and eosin (H&E) staining C. Magnified view of the H&E-stained section of the area in which spatial transcriptomics was performed for the representative WT DPM displayed in Figure 6C-D. Green: PanCK, Blue: SYTO-13 nuclear staining

D.Whole-slide GeoMx Digital Spatial Profiler (DSP) scan, E. Hematoxylin and eosin (H&E) staining F. Magnified view of the H&E-stained section of the area in which spatial transcriptomics was performed for the representative CDKN2Adel DPM displayed in Figure 6C-D. Green: PanCK, Blue: SYTO-13 nuclear staining
